# TAF1D Functions as a Novel Biomarker in Osteosarcoma

**DOI:** 10.7150/jca.85688

**Published:** 2023-07-09

**Authors:** Yu-Nan Man, Yu Sun, Pei-Jun Chen, Hao Wu, Mao-Lin He

**Affiliations:** 1 Division of Spinal Surgery, The First Affiliated Hospital of Guangxi Medical University, Shuangyong Road 6, Nanning, Guangxi Zhuang Autonomous Region, P.R. China, 530021.; 2 Guangxi Collaborative Innovation Center for Biomedicine, Guangxi Medical University, Nanning, Guangxi Zhuang Autonomous Region, P.R. China. 530021 (Guangxi-ASEAN Collaborative Innovation Center for Major Disease Prevention and Treatment, Guangxi Medical University, Nanning, Guangxi Zhuang Autonomous Region, P.R. China, 530021).

**Keywords:** TATA-box binding protein associated factor, RNA polymerase Ⅰ subunit D (TAF1D), osteosarcoma, clinical significance, biomarker.

## Abstract

**Background:** The most frequent primary bone cancer in teenagers, osteosarcoma (OS), is particularly aggressive with a high mortality rate.

**Methods:** By combining public databases, OS and non-cancer samples were obtained. The Wilcoxon test and standardized mean difference (SMD) were utilized to evaluate the mRNA expression level of TATA-box binding protein associated factor, RNA polymerase 1 subunit D (TAF1D). The potential of TAF1D to discriminate OS samples from non-cancer samples was revealed by summary receiver operating characteristic curve (sROC). To investigate the prognostic significance, Kaplan‒Meier curve and univariate Cox analysis were performed. Immunohistochemistry (IHC) was used to determine the TAF1D protein expression level. ESTIMATE algorithm and TIMER2.0 database were used to reveal the association between TAF1D expression and the immune microenvironment. Enrichment analysis and potential drug prediction were performed to clarify the underlying molecular mechanisms and possible therapeutic directions of TAF1D. Ultimately, the transcription factors (TFs) and the TAF1D binding site were predicted based on the Cistrome and JASPAR databases.

**Results:** TAF1D was upregulated in OS at the mRNA and protein levels and possessed robust discriminatory power. TAF1D upregulation was suggestive of worse prognosis and enhancement of tumor purity in OS patients. The cell cycle was the most significantly enriched pathway, and NU.1025 was considered to be the potential target agent. Finally, MYC was identified as a TF that regulates the expression of TAF1D.

**Conclusions:** Altogether, TAF1D has the potential to serve as a biological marker and therapeutic target in OS, which could offer new perspectives for OS treatment.

## Introduction

Osteosarcoma (OS) is a malignant primary tumor originating from bone tissue, and the mainstream view is that the incidence of OS shows a bimodal distribution, in which children and adolescents have the highest incidence of approximately 1-3 cases/million, followed by elderly individuals over 65 years old 1, 2. Compared with other common childhood tumors, OS has a more complex genomic mode of action and a larger number of driving genes. Some studies have shown that in patients with low-grade OS, other germline pathogenic variants of genes previously unrelated to OS can be detected, while in patients with high-grade OS, a very high somatic mutation rate is observed 3-5. According to the French Association for Cancer of Children and Adolescents research, the five-year median survival of OS patients treated with the clinically mainstream MAP chemotherapy regimen (high-dose methotrexate + doxorubicin + cisplatin) can reach 71%; however, patients with metastasis or recurrence have only a 20% survival rate 6. In addition, compared with the treatment approach of surgical resection combined with chemotherapy developed in the 1970s, the overall survival of patients with primary OS has not improved significantly 7. One of the important reasons for the above dilemma is that the histological pattern of OS is very complex and lacks specific diagnostic biomarkers 8. Therefore, it is necessary to find new biomarkers of OS for early diagnosis and ultimately improve the clinical outcome of OS patients.

TATA-box binding protein associated factor, RNA polymerase Ⅰ subunit D (TAF1D) is also called TAF(Ⅰ)41, and the coding region is located on human chromosome chr11:93736161-93739304 (https://www.genecards.org/). TATA binding protein (TBP) can bind different types of intracellular proteins to form different protein complexes, such as SL1, which is necessary for the transcription of RNA polymerase I 9. TAF1D is considered to be an important link in maintaining the transcriptional activity of the SL1 protein complex in Pol I. Therefore, its abnormal expression may lead to an imbalance in its posttranscriptional level in cells. In addition, based on single-cell sequencing analysis of 13,736 degenerated intervertebral disc cells and normal control cells and RT-qPCR experimental verification results, TAF1D was significantly overexpressed in both degenerated nuclei pulpous and degenerated intervertebral discs, and was chosen as a specific marker gene of intervertebral disc degeneration. Some studies also showed that reduced expression of TAF1D may lead to the downregulation of Pol I transcription and ultimately induce HeLa cell apoptosis and inhibit cell proliferation 10-13. In human cells, gene transcription is regulated in the cell cycle. Pol I, which is closely related to TAF1D, is inhibited in mitosis. Based on cervical cancer, studies have confirmed that G2/M-specific phosphorylation of the TAF1D site is detected, which indicates that TAF1D may play a key role in the regulation of the tumor cell cycle 10, 14, 15. It is generally believed that the disorder of cell cycle control in vivo may lead to abnormal growth of OS cells, therefore, it is interesting to explore whether TAF1D plays a role in regulating the cell cycle during the progression of OS 16. Previous research has demonstrated a substantial correlation between the poor prognosis of neuroblastoma patients and the overexpression of TAF1D 17. However, little is known about the function and role of TAF1D in other malignant tumors, especially in OS. Therefore, our study aims to clarify the clinical significance and potential molecular mechanism of TAF1D in OS.

In this study, we first summarized the pan-cancer expression of TAF1D and explored its clinical significance in multiple cancers. Then, for the first time, based on microarray and high-throughput sequencing samples, we explored the mRNA expression level, discriminative ability and prognostic value of TAF1D in OS. Tissue microarrays were used to determine the protein expression level of TAF1D in OS. Based on the expression of TAF1D, the relevance between TAF1D and the immune microenvironment of OS was also explored. Subsequently, functional enrichment analysis was used to explore the potential molecular mechanism of the involvement of TAF1D in OS. Finally, we predicted possible targeted drugs and upstream regulated transcription factors (TFs).

## Materials and methods

### Acquisition of pan-cancer and OS samples

We downloaded the standardized mRNA expression profile as well as the patient prognosis information from TCGA Pan-Cancer (n=10535) database from UCSC Xena (http://xena.ucsc.edu/). The selected sample sources are primary tumors, normal solid tissue, and primary hematogenous cancer peripheral blood 18. TPM (Transcripts Per Million) data format converted by log_2_ (x+0.001) was selected to make it close to the normal distribution for accurate statistics later. In addition, we excluded the datasets in which the number of normal control samples was less than three, subsequently, we extracted TAF1D expression data in every cancer.

Gene Expression Omnibus (GEO) database, ArrayExpress database and R2 database (https://hgserver1.amc.nl/cgi-bin/r2/main.cgi) were used to obtain TAF1D expression data and clinical information of patients in OS. The search strategy was “(osteosarcoma OR osteogenic sarcoma) and (mRNA) and (*Homo sapiens*)”. The inclusion criteria were as follows: (1) the study comprised both OS and non-cancer groups; and (2) patients received no chemotherapy, radiation, or other treatment prior to tissue collection. The exclusion criteria were as follows: (1) non-human samples or cell line samples; and (2) the study did not include the expression data of TAF1D in OS. We also used the TARGET (https://ocg.cancer.gov/programs/target) public database to obtain OS mRNA expression and outcome data to define the prognostic value of TAF1D in OS. The Human Protein Atlas (HPA) database (https://www.proteinatlas.org/) is widely used to explore the protein expression levels of target genes in tumor tissues. Therefore, in this study, it was applied to explore the protein expression level of TAF1D in a variety of malignant tumors.

### Processing of expression spectrum data

Included mRNA expression profiles collected from GEO, ArrayExpress and TARGET cohorts were annotated by the gff3 file downloaded from GENCODE (https://www.gencodegenes.org/human/). When duplicate gene names existed, the median gene expression level was included in the analysis. We also filtered the samples with an average expression level of 0 and adopted log_2_ (x+0.001) transformation for the data that were not standardized so that they approximately conformed to a normal distribution. Finally, for datasets that only provided FPKM (Fragments Per Kilobase of exon model per Million mapped fragments) data format, we converted FPKM values to TPM (Transcripts Per Kilobase of exon model per Million mapped reads) values for the subsequent analysis.

### Immunohistochemistry (IHC)

Two tissue microarrays were obtained, including 11 normal bone tissues and 50 OS tissues, and the patients consisted of 22 women and 39 men with a median age of 28. The above tissue microarrays (L1024901 and LN020Bn01) were purchased from Guanghua, Inc. (Xi 'an, China). A rabbit anti-TAF1D antibody (ab2543009, dilution 1:100) was used for the immunohistochemical (IHC) experiment. To obtain accurate results, the experiment was performed following the manufacturer's instructions. For IHC experimental processing, the evaluation and scoring criteria for staining intensity were as previously described 19.

### The mRNA expression level and clinical significance of TAF1D in 26 different cancers as well as OS

No previous study has explored the mRNA expression level and discriminative ability of TAF1D in malignant tumors. Therefore, we used the Wilcoxon test to evaluate the difference in TAF1D expression between the OS group and the non-cancer control group, which was visualized using the R package "ggplot2". The area under the curve (AUC) of the ROC curve was used to quantify the discriminative ability of TAF1D expression in cancer samples and non-cancer samples. Generally, when the AUC value is greater than 0.6 and closer to 1, it indicates that it is reliable to differentiate samples according to TAF1D expression. In addition, we used Stata15.0 software to combine the mRNA expression of TAF1D in all OS datasets to explore the overall expression level after selecting an accurate model for evaluation according to I^2^ value and *p* value output by Cochran's Q test. If the study did not have high heterogeneity (I^2^<50%, *p*>0.05), a fixed effect model was selected. Similarly, the ROC curves of TAF1D in each OS dataset were merged to generate summary receiver operating characteristic (SROC) curves for the overall assessment of discrimination.

The association between TAF1D expression and patient prognosis was explored using Kaplan‒Meier curves and univariate Cox analysis. In addition, as a potential biomarker, it was necessary to explore whether TAF1D has the ability to identify OS early. Therefore, we used the R packages "survival" to calculate the association between TAF1D diagnostic ability and the time of onset of OS patients and to generate output time-dependent AUC curves 20, 21.

### The relationship between the expression level of TAF1D and the immune microenvironment of OS

The immune microenvironment (IME) constructed by malignant solid tumor tissues includes not only tumor cells but also immune cells, stromal cells, and fibroblast cells. These cells are believed to play an important role in tumor growth, disease progression, and drug resistance 22. The ESTIMATE algorithm, which uses the mRNA expression profiling of tumors to estimate stromal and immune cell content and calculate immune scores, stromal scores, and tumor purity, has been applied several times 23. In addition, the TIMER2.0 database (http://timer.comp-genomics.org/) was also used to predict the infiltration of immune cells in the OS microenvironment 24, 25. In this study, we used the ESTIMATE algorithm and TIMER2.0 database to describe the overall level of the OS immune microenvironment, after which we calculated the correlation between TAF1D mRNA expression and immune cell infiltration levels as well as the immune relevant score to explore the function that TAF1D plays in the OS microenvironment.

### Identification of TAF1D co-expressed genes in OS

It is generally believed that co-expression between genes is strong evidence of coregulation and functional correlation 26. TAF1D co-expressed genes were identified from TAF1D positive relative-genes (TAF1D-RGs) and upregulated differentially expressed genes (up-DEGs) in datasets that contained more than three non-cancer samples. The correlation between gene expression and TAF1D expression was examined in each dataset, and genes with Pearson's r≥0.3 and *p*<0.05 in at least three datasets were identified as TAF1D-RGs. Based on microarray as well as RNA sequencing datasets, DEGs were identified using the limma and limma-voom method 27, 28. In addition, we also used the RobustRankAggreg (RRA) algorithm to select the differentially expressed genes that were upregulated in all datasets and defined them as up-DEGs 29. Only the genes appearing in TAF1D-RGs and up-DEGs were considered to possibly interact with TAF1D.

### Functional enrichment analysis

The R package 'Cluster profiler' was used to perform KEGG pathway analysis. Gene‒gene interrelation and functions were explored through the Gene MANIA database (http://genemania.org/), which is based on the TAF1D co-expressed genes. Using the STRING database (https://string-db.org/), a PPI network of TAF1D co-expressed genes was formed. After that, the Cytohubba plug-in of Cytoscope 3.8.0 was used to screen hub genes of the PPI network.

### Prediction of targeting TAF1D drugs

For a potential biomarker of OS, it is necessary to explore effective targeted drugs. According to the Library of Integrated Network based Cellular Signatures (LINCS) database, the eXtreme Sum (XSum) method is considered to have the best performance in drug evaluation and has been confirmed in the treatment of liver cancer 30. To conduct a difference analysis, we split the patients into two groups based on the median TAF1D expression: the high TAF1D expression group and the low TAF1D expression group. We integrated the grouping information and gene expression profile and calculated the potential drug score through the R packages "PharmacoGx" and "pRRophetic" 31. The lower the score, the more likely the drug is to reverse the molecular characteristics of the disease, and the more likely it is to have the ability to treat the disease.

### The potential upstream transcription factor of TAF1D

Cistrome Data Browse (http://cistrome.org/db/#/) is a database that combines experimental data of chromatin immunoprecipitation sequences (ChIP-seq) to predict the TFs of target genes 32. Therefore, it was used to predict potential TF binding to the promoter region of TAF1D (based on a 1 kb sequence upstream of the transcription start site); before that, we defined the biological source as OS cell lines. The possible regulated TFs should be upregulated in OS and positively correlated with TAF1D. Finally, through the JASPAR database (https://jaspar.genereg.net/), we explored the binding motif of the TAF1D promoter region and the predicted TF 33.

### Statistical analysis

In this study, STATA 15.0 and R 4.1.0 software were used for statistical analysis and chart drawing. When the associated 95% confidence interval (CI) for the SMD value did not contain 0, the results were considered clinically meaningful. The robustness of each dataset analyzed in the meta-analysis was evaluated using sensitivity analysis. To determine whether there was publication bias in the included studies, Deek's test and Begg's test were applied. If not specifically mentioned, *p*<0.05 was used to determine statistical significance.

## Results

### The mRNA expression level of TAF1D in cancers

**Figure 1:**
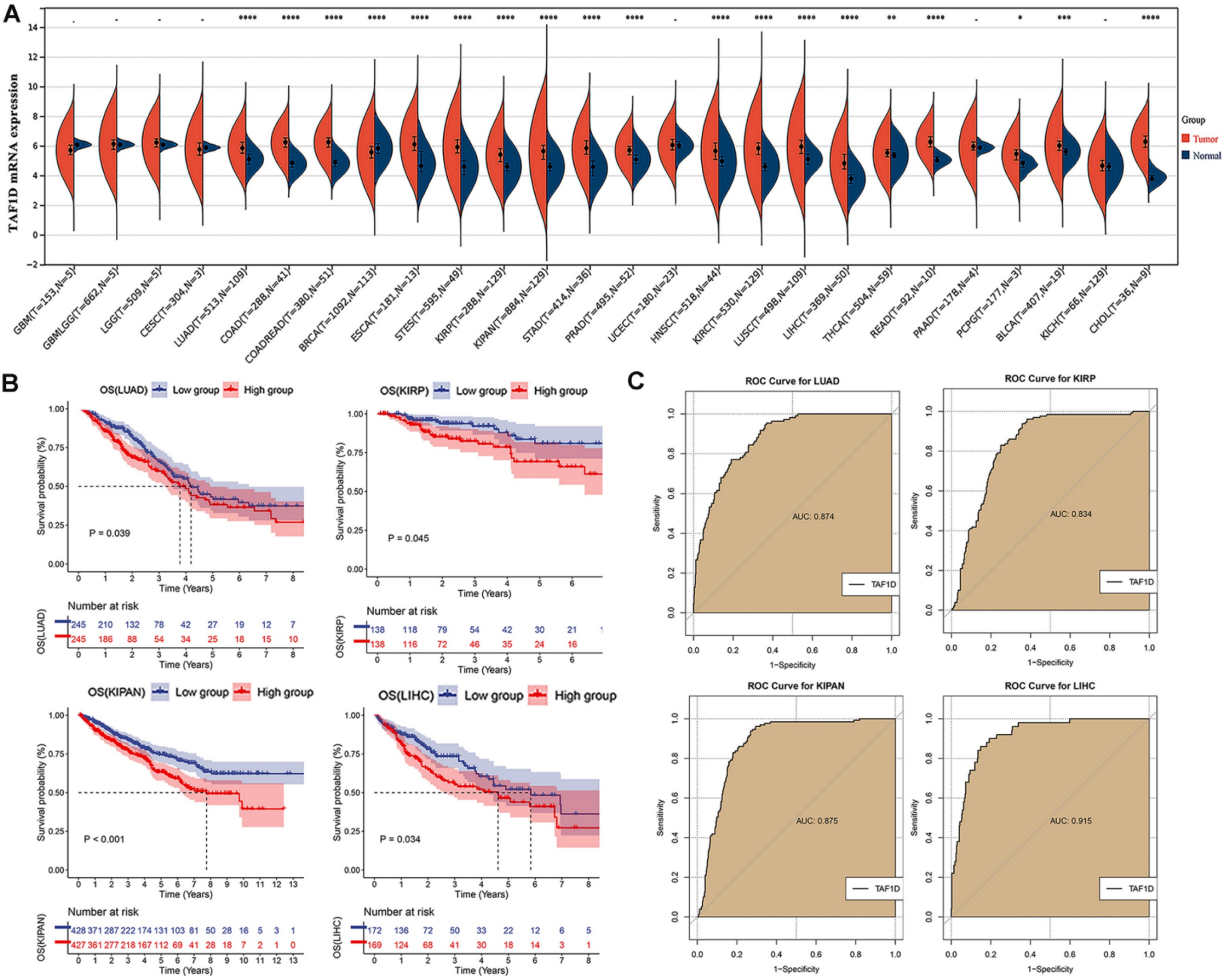
** The mRNA expression level, prognostic and discriminative significance of TAF1D in cancers.** (A) TAF1D mRNA expression level in 26 different cancers. (B) TAF1D high expression was related to shorter survival time of LUAD, KIRP, KIPAN and LIHC patients. (C) ROC curves of TAF1D in LUAD, KIRP, KIPAN and LIHC. *p <0.05; **p <0.01; ***p<0.001; ****p<0.0001.

**Figure 2:**
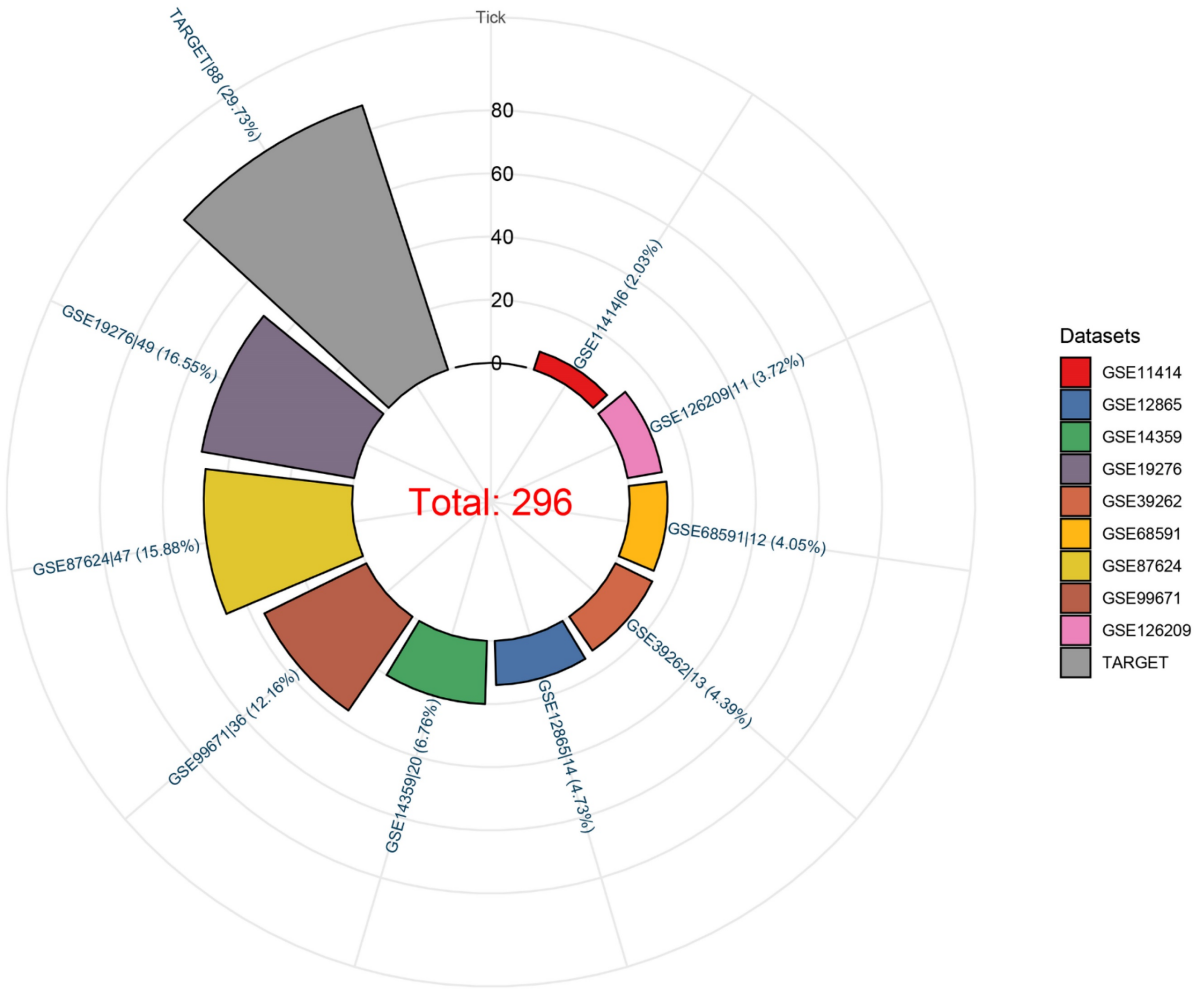
Datasets included in the study and their sample numbers.

**Figure 3:**
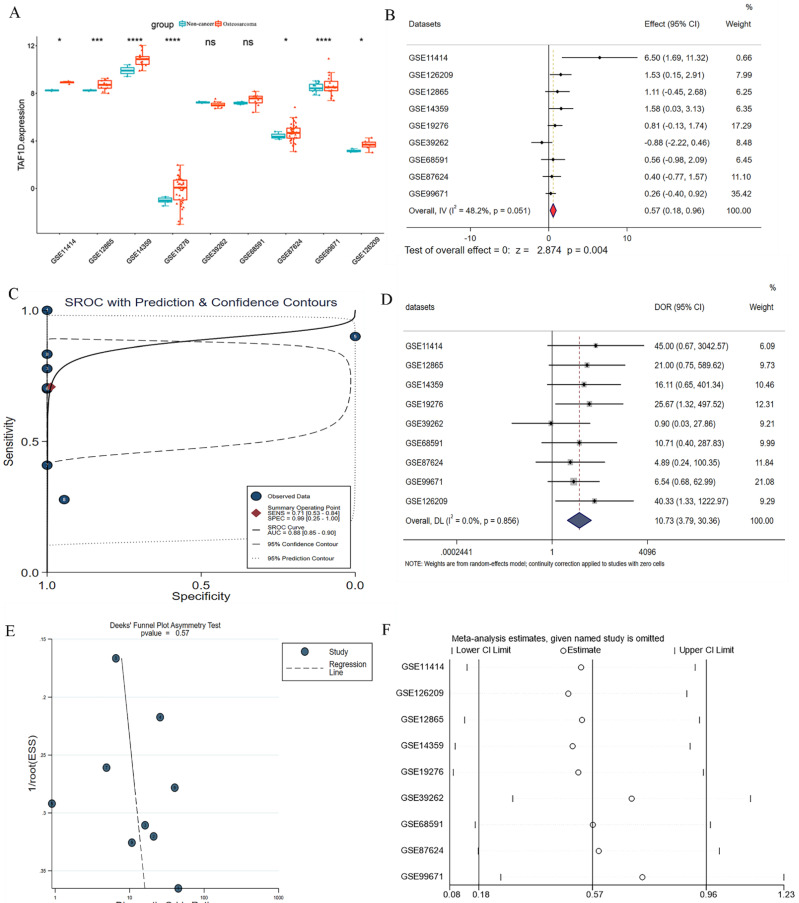
** The mRNA expression level and discrimination potential TAF1D in OS.** (A) Box plots of TAF1D expression in OS. (B) Forest diagram of TAF1D mRNA expression in OS. (C) SROC curve assessing the discrimination potential of TAF1D in OS. (D) Forest plot of the diagnostic value of TAF1D in OS. (E) Deek's funnel diagram, which suggested no publication bias (*p* > 0.05). (F) Sensitivity analysis of TAF1D expression in OS.

We observed that the mRNA expression level of TAF1D was considerably elevated in 19 cancers by analyzing the TCGA Pan-Cancer dataset (Fig. 1A), similarly, we also investigated the protein expression levels of TAF1D in various malignant tumors, which showed that breast, ovarian, cervical, skin, pancreatic, urothelial, lung, and stomach cancers displayed strong or moderate cytoplasmic and membranous positivity, indicating a high protein expression level of TAF1D in these cancers (Fig. S1). Moreover, the findings of the prognostic significance and discriminative power of TAF1D across cancers indicated that in four cancers, LUAD, KIRP, KIPAN and LIHC, the upregulation of TAF1D was closely related to poor prognosis and had good discriminatory potential, especially in LIHC (AUC=0.915), which indicates that TAF1D may be crucial to the development of the aforementioned malignancies (Fig. 1 B-C). Additionally, the ROC curves of the other cancer samples were also sequentially plotted to explore the discriminating ability of TAFD in malignancy (Fig. S2). Since the TCGA Pan-Cancer dataset does not contain OS samples, we collected 9 GEO datasets and TARGET datasets that met the inclusion and exclusion criteria, including 254 OS samples and 42 non-cancer control samples. The number of samples in each dataset is displayed in Fig. 2. In seven datasets, the Wilcoxon test revealed that the level of TAF1D mRNA expression was substantially higher in OS samples than in control samples; however, there was no significant difference in the GSE39262 and GSE68591 datasets (Fig. 3A). Therefore, we integrated the expression of TAF1D into the above datasets. The results showed that the mRNA expression level of TAF1D in OS samples was higher than that in non-cancer control samples (SMD=0.57, 95% CI [0.18-0.96]) (Fig. 3B). In addition, for each dataset, we also drew ROC curves to evaluate the differentiation ability of TAF1D in OS and integrated all ROC curves to finally obtain sROC curves and calculate the diagnostic odds ratio (DOR). The results showed that TAF1D has good differentiation potential for OS (AUC=0.88, 95% CI [0.85-0.90], DOR=10.73, 95% CI [3.79-30.36]) (Fig. 3C-D and Fig. S3). Finally, we strictly evaluated the quality of the included databases. The results of Deek's and Begg's tests showed that our research had no significant publication bias (Fig. 3E and Fig. S4). Sensitivity analysis showed that no dataset was a source of heterogeneity (Fig. 3F).

### The clinical significance of TAF1D in OS patients

**Figure 4:**
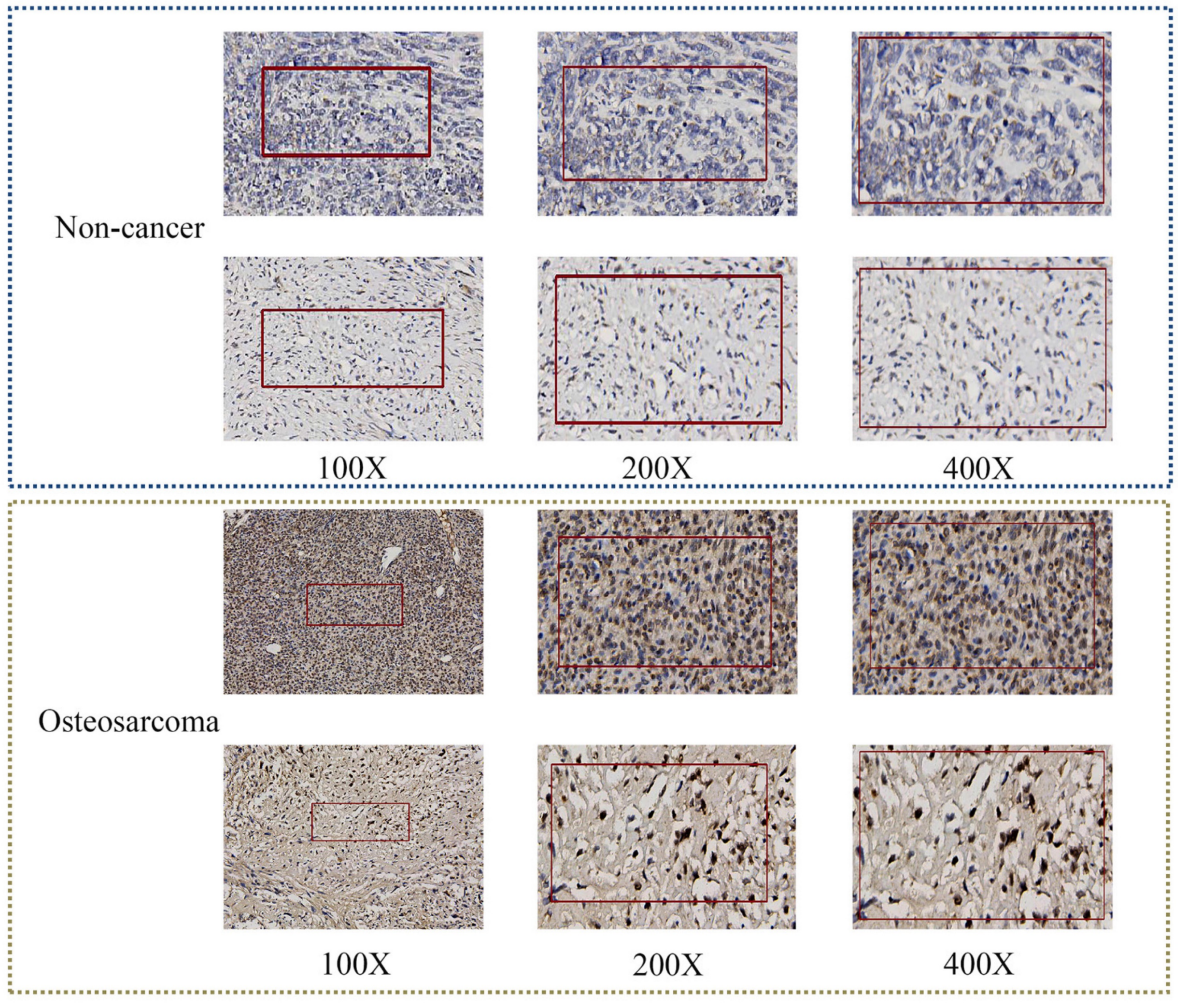
** TAF1D protein levels in OS tissues by IHC**. TAF1D expression was intense in OS tissues compared to normal tissues (magnification, x100 left, x200 middle and x400 right).

**Figure 5:**
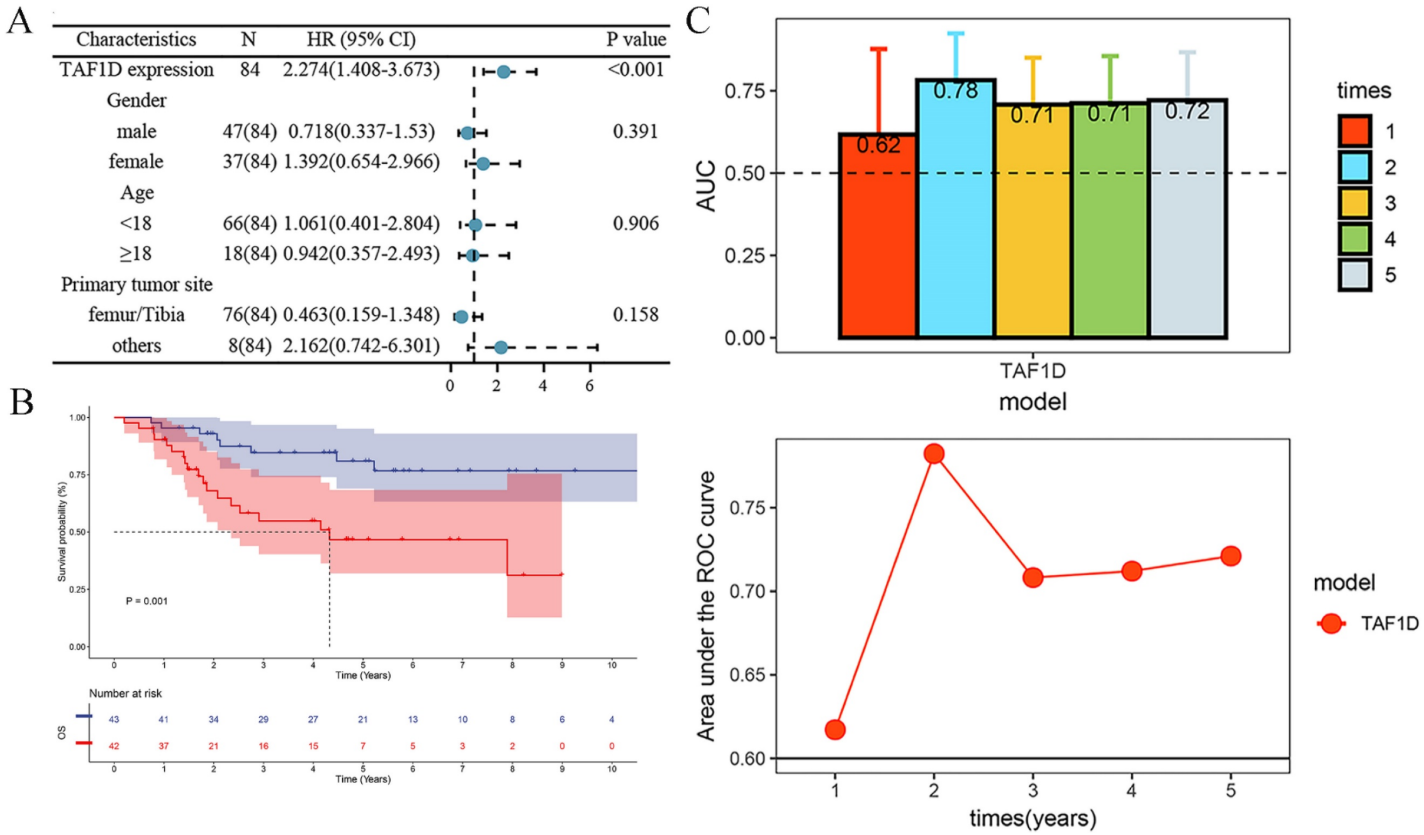
** Relationship of TAF1D expression with prognosis of OS patients.** (A) Univariate Cox analysis demonstrates TAF1D expression as a risk factor for OS patients based on TARGET dataset. (B) TAF1D high expression was related to shorter survival time of OS patients based on TARGET dataset. (C) Time-dependent AUC of TAF1D expression based on TARGET dataset.

After confirming that the mRNA expression level of TAF1D was higher in OS samples than in control samples, we explored the protein level of TAF1D. The IHC results showed that TAF1D was significantly upregulated in OS (Fig. 4 and Fig. S5). After integrating the expression amount and prognostic information of TAF1D in the TARGET database, we conducted univariate Cox and Kaplan‒Meier survival analyses and their clinical features. The results showed that when TAF1D is highly expressed, it often indicates a poor prognosis of OS patients. Compared with other clinical characteristics (sex, age, and primary tumor site), TAF1D was the only factor that indicated a poor prognosis (Fig. 5A-B). The *t*-test results indicate that the expression level of TAF1D is closely related to the survival status of patients with OS, but not significantly correlated with other clinical states (Table 1). Based on multiple statistical testing methods, the data further demonstrate that TAF1D is closely associated with poor prognosis in patients with OS. More recently, we explored the predictive ability of TAF1D in relation to the time of illness, and the results showed that TAF1D had the highest predictive ability (AUC = 0.78) in the first 1-2 years of illness. The above results indicated that TAF1D may have the potential to serve as an early prognostic marker of OS (Fig. 5C).

### The relationship between the TAF1D expression level and the OS immune microenvironment

**Figure 6:**
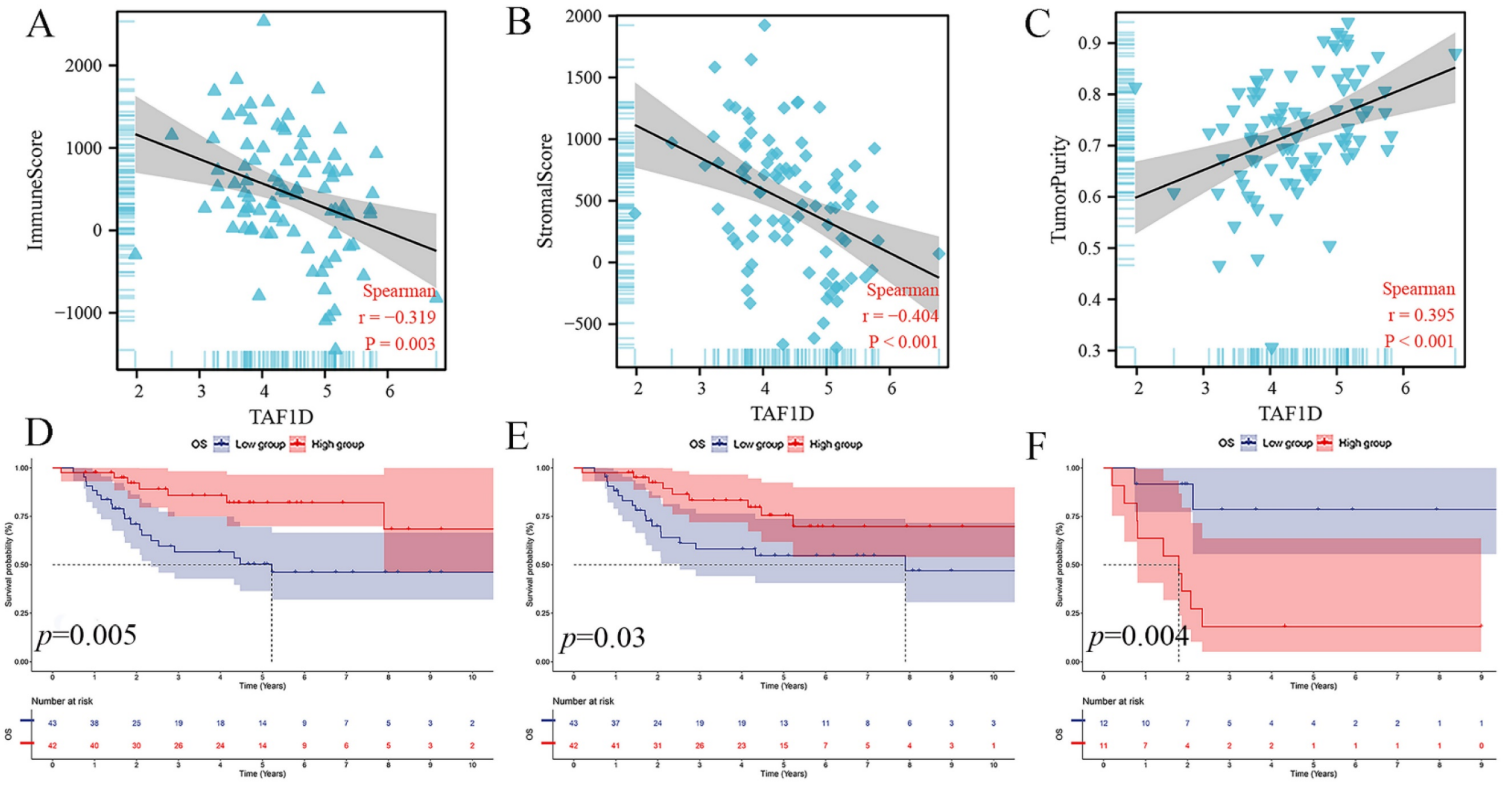
** Relationship of TAF1D expression with the IME in OS.** (A-C) Correlation between TAF1D expression and Immune score and Stromal score and Tumor purity using ESTIMATE algorithm. (D-E) Immune and Stromal low scores were related to shorter survival time of primary OS patients. (F) Enhancement of Tumor purity was related to shorter survival time of metastasis OS patients.

The ESTIMATE algorithm showed that the TAF1D expression level was negatively correlated with the immune score and stromal score (r=-0.319, r=-0.404) but positively correlated with tumor purity (r=0.395) (Fig. 6A-C). In addition, based on the median immune score and stromal score, we divided OS patients into high-score and low-score groups and carried out survival analysis. The results showed that patients with higher immune or stromal scores had a better prognosis (Fig. 6D-E). Furthermore, we also divided patients with OS metastasis into two groups based on the tumor purity score. The results showed that patients with bone metastasis with higher tumor purity had a worse prognosis (Fig. 6F).

**Figure 7:**
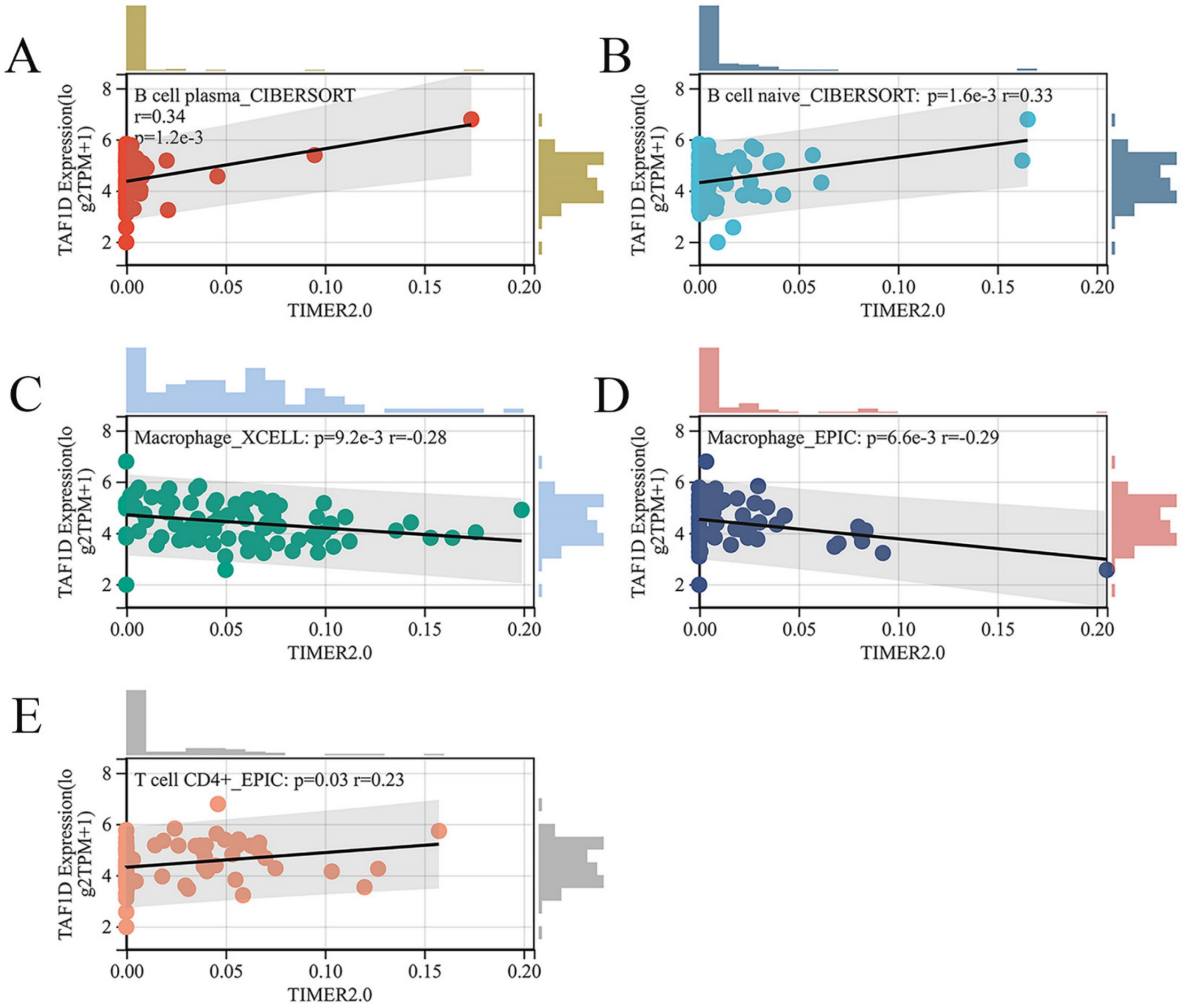
** Relationship of TAF1D expression with the infiltration of various immune cells based on the TIMER2.0 database.** (A-B) Correlation between TAF1D expression and the degree of B cells infiltration. (C-D) Correlation between TAF1D expression and the degree of Macrophage cells infiltration. (E) Correlation between TAF1D expression and the degree of and CD4+ T cells.

Subsequently, we explored the relationship between the infiltration of various immune cells and the expression of TAF1D in the OS immune microenvironment through the TIMER2.0 database (Fig. S6). The findings demonstrated that the degree of B cells and CD4^+^ T cells infiltration was generally positively correlated with TAF1D expression level, while the degree of Macrophage infiltration was negatively correlated with TAF1D expression level (Fig. 7A-E).

### Exploration of the potential molecular mechanism of TAF1D in OS

**Figure 8:**
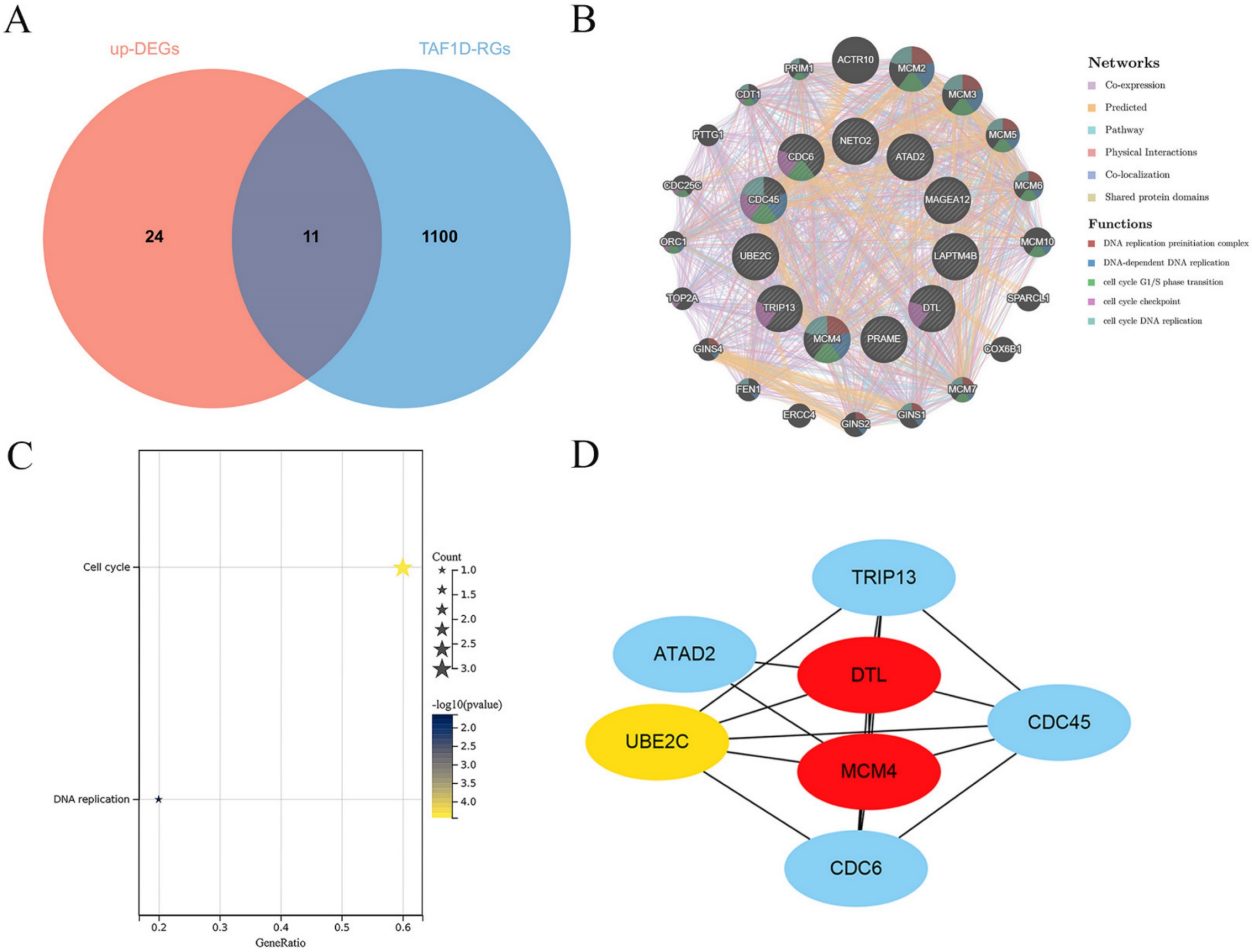
** Exploration of the potential molecular mechanism of TAF1D in OS.** (A) Venn diagram showing the TAF1D co-expressed genes in OS based on up-DEGs and TAF1D-RGs. (B) Gene-gene interactions of TAF1D co-expressed genes based on Gene MANIA database. (C) Enrichment terms of TAF1D co-expressed genes in KEGG pathway. (D) PPI network analysis showed that DTL and MCM4 had the top two degrees of connection of the TAF1D co-expressed genes.

Through correlation analysis in the included datasets, we screened 1111 TAF1D-RGs and obtained 35 significantly upregulated DEGs through the RRA algorithm (Fig. S7 and Table S1). A total of 11 co-expressed TAF1D genes were obtained after intersection (Fig. 8A). The analysis results of the GeneMANIA database showed that the 11 TAF1D co-expressed genes obtained are closely related to protein domains, and their biological functions are also closely related to “DNA replication” and “cell cycle” (Fig. 8B). The enrichment analysis of the above co-expressed genes is helpful to reveal the potential molecular mechanism of TAF1D in OS. The KEGG enrichment results showed that "cell cycle" and "DNA replication" were significantly enriched pathways (Fig. 8C). In addition, the PPI network showed that DTL and MCM4 may be hub genes, and they are also considered to be most likely to interact with TAF1D to jointly promote the development of OS (Fig. 8D).

### Potential therapeutic drugs targeting TAF1D for OS

**Figure 9:**
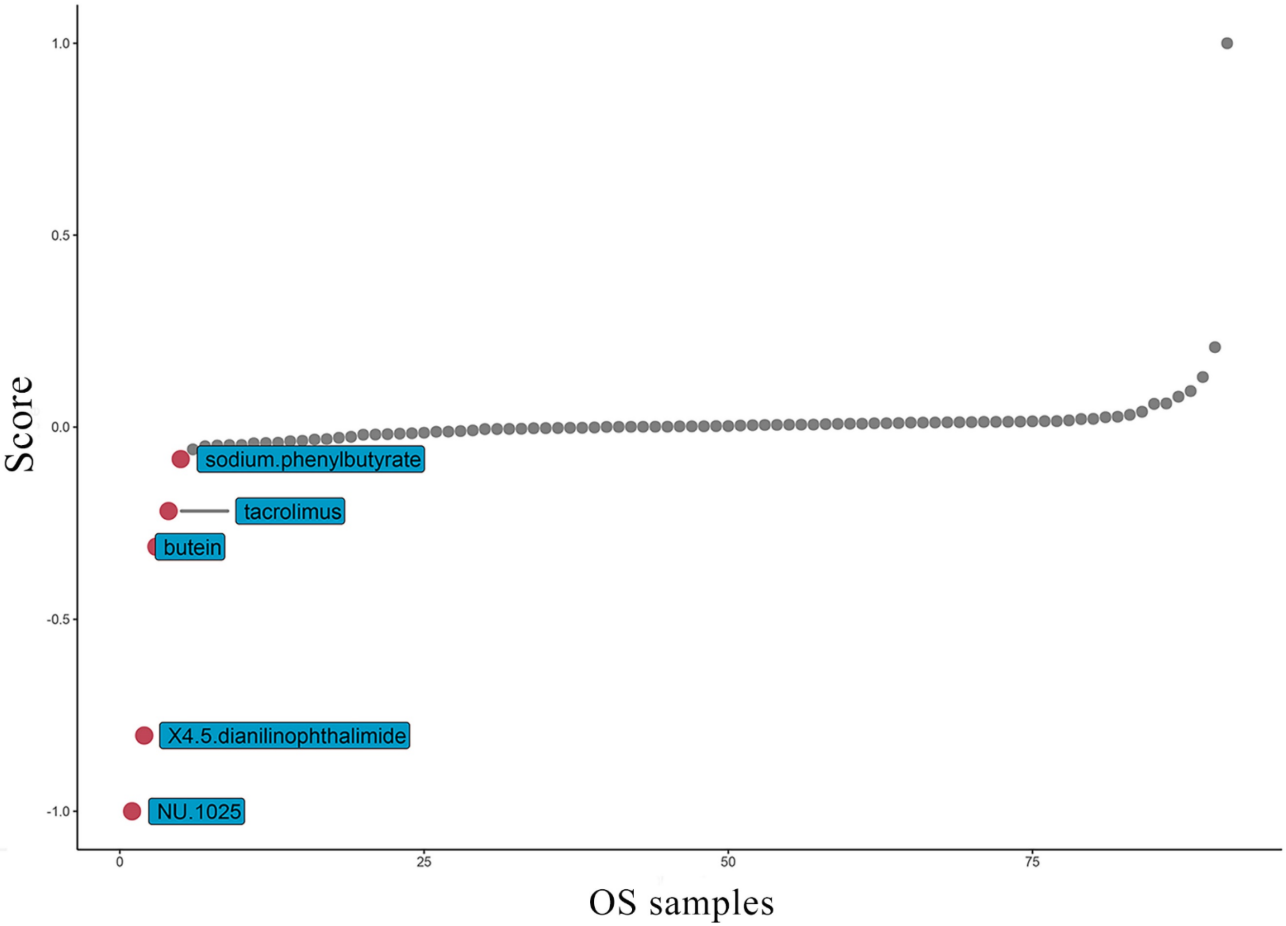
Top five most potential drugs targeting TAF1D for OS treatment.

At present, the common drug used to treat OS is methotrexate. Grouping based on the amount of TAF1D expression, the results suggest that patients with high TAF1D expression have higher drug sensitivity to methotrexate (Fig. S8). However, in view of the low survival rate of patients with bone metastasis, it is necessary to find new targeted drugs. Therefore, based on the "PharmacoGx" R package, we found that NU.1025 has the most significant drug sensitivity to patients with high TAF1D expression and was also considered to be a drug that may target TAF1D (Fig. 9).

### TAF1D potential upstream transcription factor

**Figure 10:**
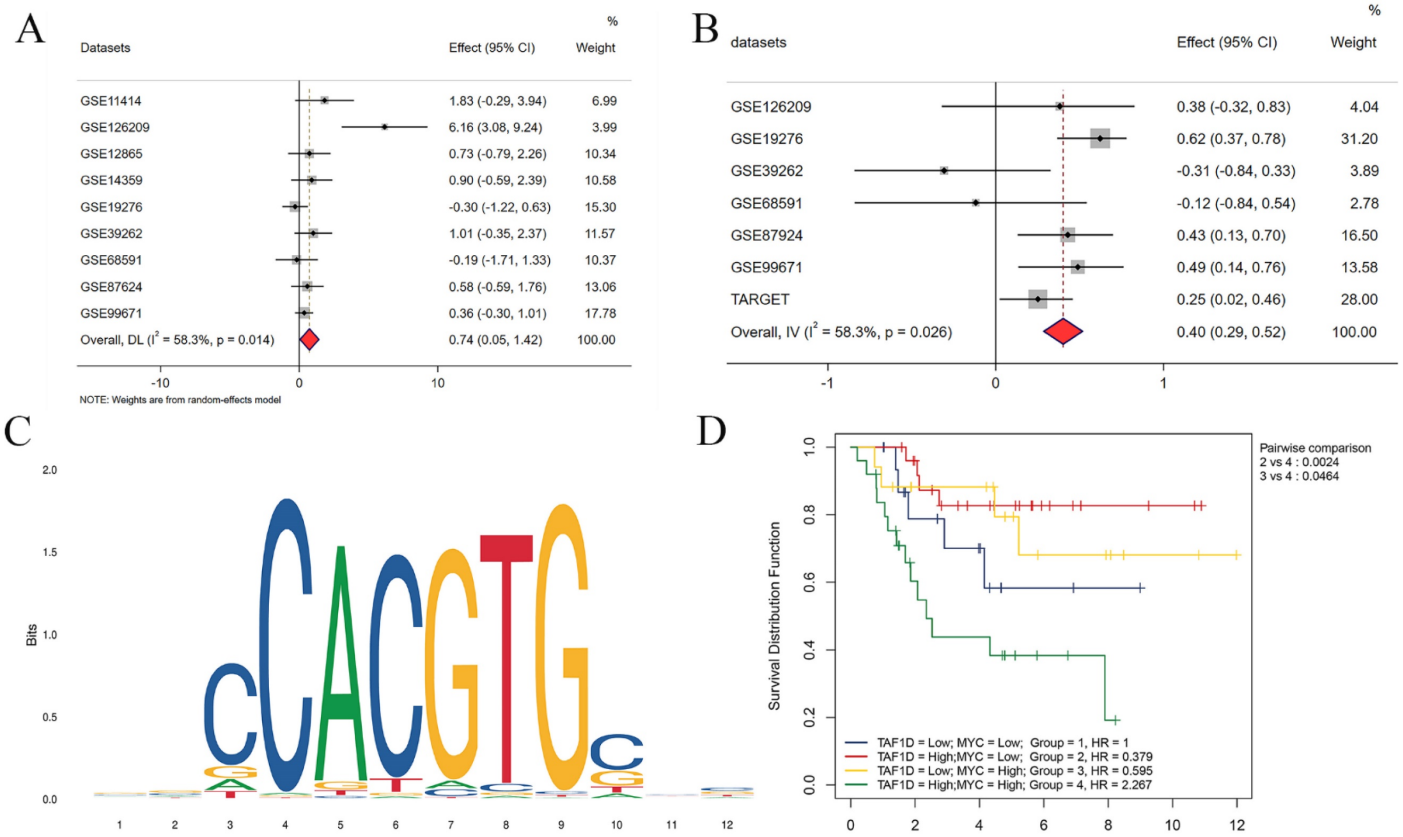
** MYC has the potential to serve as the upstream transcription factor of TAF1D.** (A) Forest diagram of MYC mRNA expression in OS. (B) An integrative analysis of correlation analysis between TAF1D and MYC. (C) The binding site of MYC in the promoter region of TAF1D. (D) Kaplan-Meier curve based on TAF1D and MYC expression together.

Our above results indicate that TAF1D is highly expressed in OS and is associated with poor prognosis, so it is necessary to explore its underlying regulatory mechanism. After the intersection of potential TFs predicted by the Cistrome database with genes upregulated in OS (SMD>1) was generated, MYC (MYC Proto-Oncogene, BHLH Transcription Factor) was selected as a possible TF for TAF1D. The results showed that MYC was upregulated in OS samples (SMD=0.74, 95% CI [0.05-1.42]) and positively correlated with TAF1D expression (Fig. 10A-B). Similarly, based on the R2 database, we explored the methylation level of MYC, and the results showed that it was also significantly positively correlated with TAF1D expression (Fig. S9). In addition, we also predicted the sequence of the upstream region of the TAF1D promoter and MYC binding site, and the motif with the strongest correlation was GCCCACGTGGCC (Fig. 10C and Table 2). We also found that MYC has a significant binding peak at the TAF1D translation start site (Fig. S10). More recently, we investigated MYC's prognostic importance in OS patients, and the findings revealed that increased MYC expression also indicated a poor prognosis (Fig. S11). It is worth noting that patients with high expression of both TAF1D and MYC appear to have a worse prognosis than those with high expression of only one or the other, according to the findings of the two-factor survival study (Fig. 10D).

## Discussion

According to the statistical data of the American Cancer Society (ACS) in 2021, OS accounts for 3% of all childhood cancers and is the third most common cancer among children and adolescents, second only to lymphoma and brain cancer 34, 35. In clinical application, serum alkaline phosphatase (ALP) can be used as a diagnostic and more reliable prognostic tool for adult OS 36-38. However, as the main prevalence group of OS patients, the serum ALP level of children and adolescents has no specificity for the diagnosis of OS, especially when the liver function of patients is impaired, and the false-positive rate of diagnosis by serum ALP level is high 39, 40. Therefore, it is necessary to find new potential biomarkers for the early diagnosis and treatment of OS. Studies have shown that TAF1D is related to the poor prognosis of some patients with malignant tumors. However, no study has reported the relationship between TAF1D and OS.

In this study, based on public databases and IHC experiments, we confirmed for the first time that TAF1D is significantly upregulated in OS. The sROC results showed that TAF1D has the ability to be a biological marker of OS and has moderate diagnostic potential (AUC=0.88). Through the integrated analysis of TAF1D's predictive ability and the time of onset, we found that TAF1D has a certain ability to identify OS at an early stage, especially in the first to second years of onset. Given the high misdiagnosis rate of OS at an early stage, our research may provide some help for early clinical diagnosis 41. In addition, according to Cox regression analysis and the log-rank test, the overexpression of TAF1D is related to the poor prognosis of OS patients. Combined with its good discriminative ability, the results showed that TAF1D has the ability to be a clinical marker of OS.

Based on the ESTIMATE algorithm, we found that the overexpression of TAF1D was accompanied by changes in the OS microenvironment, which may indicate that in the OS microenvironment, the overall level of immune cells and stromal cells may decline with increasing TAF1D expression. Survival analysis based on immune and stromal scores showed that patients with lower scores had a worse prognosis, which may indicate that the increase in TAF1D expression accompanied by changes in the tumor microenvironment can affect the prognosis of patients with OS. Additionally, the outcomes demonstrated a strong and favorable correlation between the tumor purity score and TAF1D expression (r=0.395, p<0.001), which indicated that the increase in TAF1D expression was accompanied by the enhancement of the purity of solid OS tumors. In addition, in patients with bone metastasis, patients with high tumor purity tended to have a worse prognosis, suggesting that the increase in TAF1D expression might promote the distant metastasis of OS, but further research is needed to confirm this finding.

Additionally, the assessment of the degree of immune cell infiltration revealed a favorable correlation between the expression of TAF1D and the degree of B-cells and CD4^+^ T-cells infiltration. Studies have shown that B cells can kill tumor cells by encouraging T cells to become activated during an immune response, thus inhibiting the progression of tumors, and newer studies have shown that CD4^+^ T cells can directly play an antitumor role by inhibiting the cancer cell cycle 42, 43. In the enrichment analysis results of this study, we found that the most significant enriched pathway of TAF1D co-expressed genes was the “cell cycle pathway”, which was also confirmed by the analysis results of the GeneMANIA database. Therefore, we speculate that the process by which TAF1D promotes the progression of OS is accompanied by an increase in the infiltration of B cells and CD4^+^ T cells. When the above immune cells are depleted, they are accompanied by an imbalance in the immune microenvironment of OS.

In the present study, we revealed that NU.1025 is a possible target drug for TAF1D. NU.1025 is an effective PARP inhibitor that is believed to enhance the cytotoxicity of ionizing radiation and anticancer drugs, protect the nerves and reduce the degree of neuroinflammation 44, 45. In previous studies, NU.1025 has been proven to inhibit the proliferation of breast cancer cells and promote apoptosis and the potential as targeted therapy for thyroid adenocarcinoma 46, 47. In addition, NU.1025 is believed to enhance the cytotoxicity of the DNA methylation agents MTIC and bleomycin to enhance the anticancer effect 48. The results of our study show that patients with TAF1D overexpression are more sensitive to the commonly used clinical drug methotrexate, which indicates that TAF1D, as a potential biological marker of OS, has certain clinical application value in targeted therapy; thus, whether the combination of NU.1025 and methotrexate would enhance the efficacy of OS treatment is of great interest and requires in-depth exploration.

Previous studies have confirmed that overexpression or amplification of MYC is present in a variety of human malignancies, including OS, and is able to promote OS proliferation and metastasis 49, 50. In our study, we believe that MYC may regulate the transcriptional expression of TAF1D to promote the progression of OS. To verify our hypothesis, we carried out correlation verification at the mRNA and methylation levels and predicted the binding site of the promoter through the JASPAR website (relative score=0.96). It is worth noting that, compared with OS patients with high expression of either MYC or TAF1D, OS patients with overexpression of both MYC and TAF1D have the worst prognosis. Studies have confirmed that TAF1D can be highly co-expressed with SNHG1 (a lncRNA) to jointly promote the progression of neuroblastoma, which indicates that TAF1D may play a role in accelerating and promoting the progression of malignant tumors 16.

Of course, there are some deficiencies in our research. First, due to the lack of sufficient samples, we hope to carry out more clinical follow-up and investigation on OS patients to better evaluate the role of TAF1D in the early diagnosis of OS. Second, the predicted targeted drugs were determined by bioinformatics analysis alone; thus, further verification by performing in vitro and in vivo experiments is necessary. Finally, whether TAF1D binds to the transcription factor MYC directly or forms a transcription complex before binding to MYC needs to be determined.

In conclusion**,** our results indicate that TAF1D has clinically meaningful significance, particularly in OS, based on the study from pan-cancer and OS. TAF1D is significantly upregulated and has robust differentiation potential and early diagnostic ability, closely related to the immune microenvironment of OS. In terms of the molecular mechanism, TAF1D may be regulated by the transcription factor MYC and may play a role in the cell cycle of OS. Moreover, NU.1025 is a possible target drug of TAF1D.

## Supplementary Material

Supplementary figures and table.

## Figures and Tables

**Table 1:** Association between TAF1D expression and clinicopathological parameters in OS samples based on TARGET database

Clinicopathological		TAF1D	expression	t-test	
parameters	N	M	SD	t-value	*p*-value
**Gender**					
Female	37	2.965	0.747	0.694	0.489
Male	47	2.861	0.625		
**Age**					
<18	66	2.944	0.669	-0.976	0.332
≥18	18	2.768	0.716		
**Recurrence**					
No	65	2.867	0.645	0.991	0.325
Yes	19	3.042	0.789		
**Metastasis**					
No	63	2.869	0.706	1.128	0.263
Yes	21	3.018	0.594		
**Survival state**					
Alive	57	2.745	0.653	3.21	0.002
Dead	27	3.237	0.624		
**Primary tumor site**					
Femur/Tibia	76	2.927	0.667	0.846	0.400
Others	8	2.713	0.815		

TARGET: Therapeutically Applicable Research to Generate Effective Treatments; M: mean; N: number; OS: osteosarcoma; SD: standard deviation.

**Table 2:** The binding site of MYC in the promoter region of TAF1D and the first eight binding sequences

Name	Score	Relative score	Start	End	Strand	Predicted sequence
MA0147.3.MYC	14.25	0.97	471	482	+	GCCCACGTGGCC
MA0147.3.MYC	14.15	0.96	471	482	-	GGCCACGTGGGC
MA0147.3.MYC	9.02	0.86	229	240	-	AGCCGCGTGGCG
MA0147.3.MYC	8.95	0.85	229	240	+	CGCCACGCGGCT
MA0147.3.MYC	7.89	0.83	1267	1278	-	GGCAACGTGGTG
MA0147.3.MYC	7.38	0.82	1267	1278	+	CACCACGTTGCC
MA0147.3.MYC	7.35	0.82	2120	2131	+	GACCATGTGACA
MA0147.3.MYC	7.01	0.81	1988	1999	+	GACCACGTTCAA

TAF1D: TATA-box binding protein associated factor, RNA polymerase Ⅰ subunit D; MYC: MYC Proto-Oncogene, BHLH Transcription Factor.

## Abbreviations

OS: osteosarcoma
ROC: receiver operating characteristic
SROC: summarized receiver operating characteristic
IHC: immunohistochemistry
TARGET: Therapeutically Applicable Research To Generate Effective Treatments
GEO: Gene Expression Omnibus
AUC: area under the curve
IME: immune microenvironment
KEGG: Kyoto Encyclopedia of Genes and Genomes
PPI: protein-protein interaction
DEGs: differentially expressed genes
RRA: RobustRankAggreg
LUAD: Lung adenocarcinoma
KIRP: Kidney renal papillary cell carcinoma
KIPAN: Pan-kidney cohort (KICH+KIRC+KIRP)
LIHC: Liver hepatocellular carcinoma
