# Supplementary material for: TAF1D Functions as a Novel Biomarker in Osteosarcoma
Source: J Cancer. 2023 Jul 9;14(11):2051–65. doi: 10.7150/jca.85688 (PMC10367927; doi:10.7150/jca.85688)
Supplement: Supplementary file 1 — Supplementary figures and table. [file jcav14p2051s1.pdf]

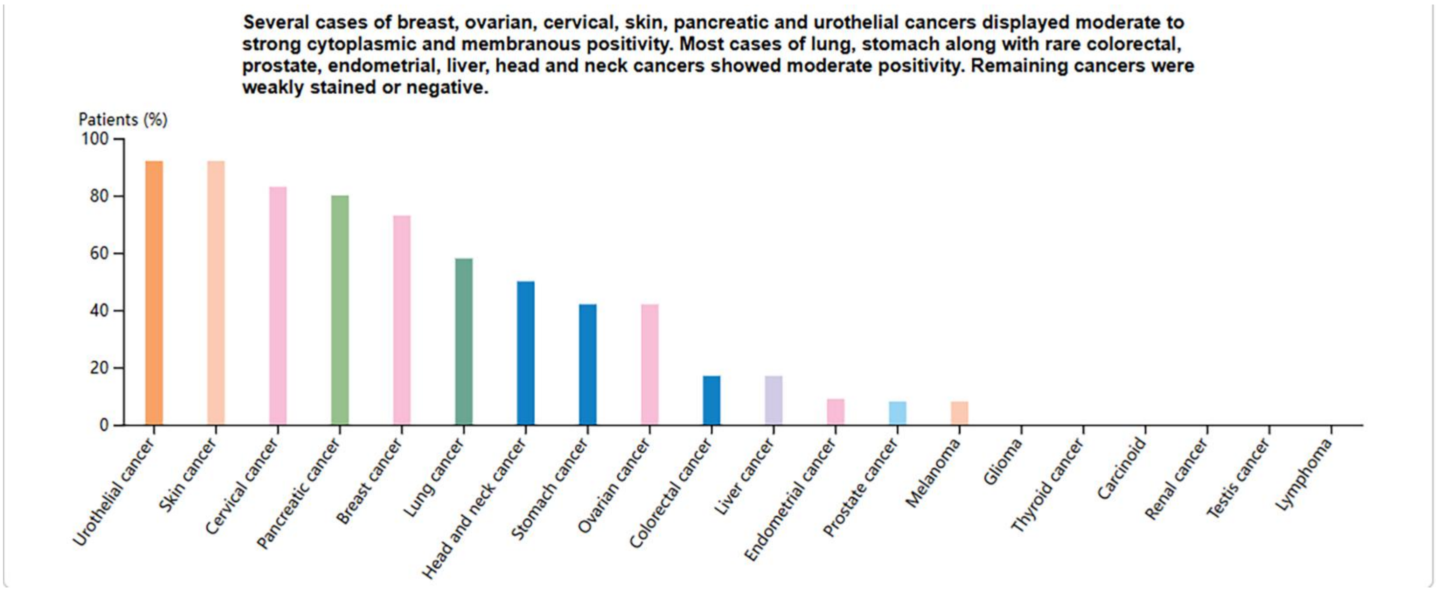

Additional Fig. Supplementary 1. The protein expression level of TAF1D in various malignant tumors, based on the HPA database.

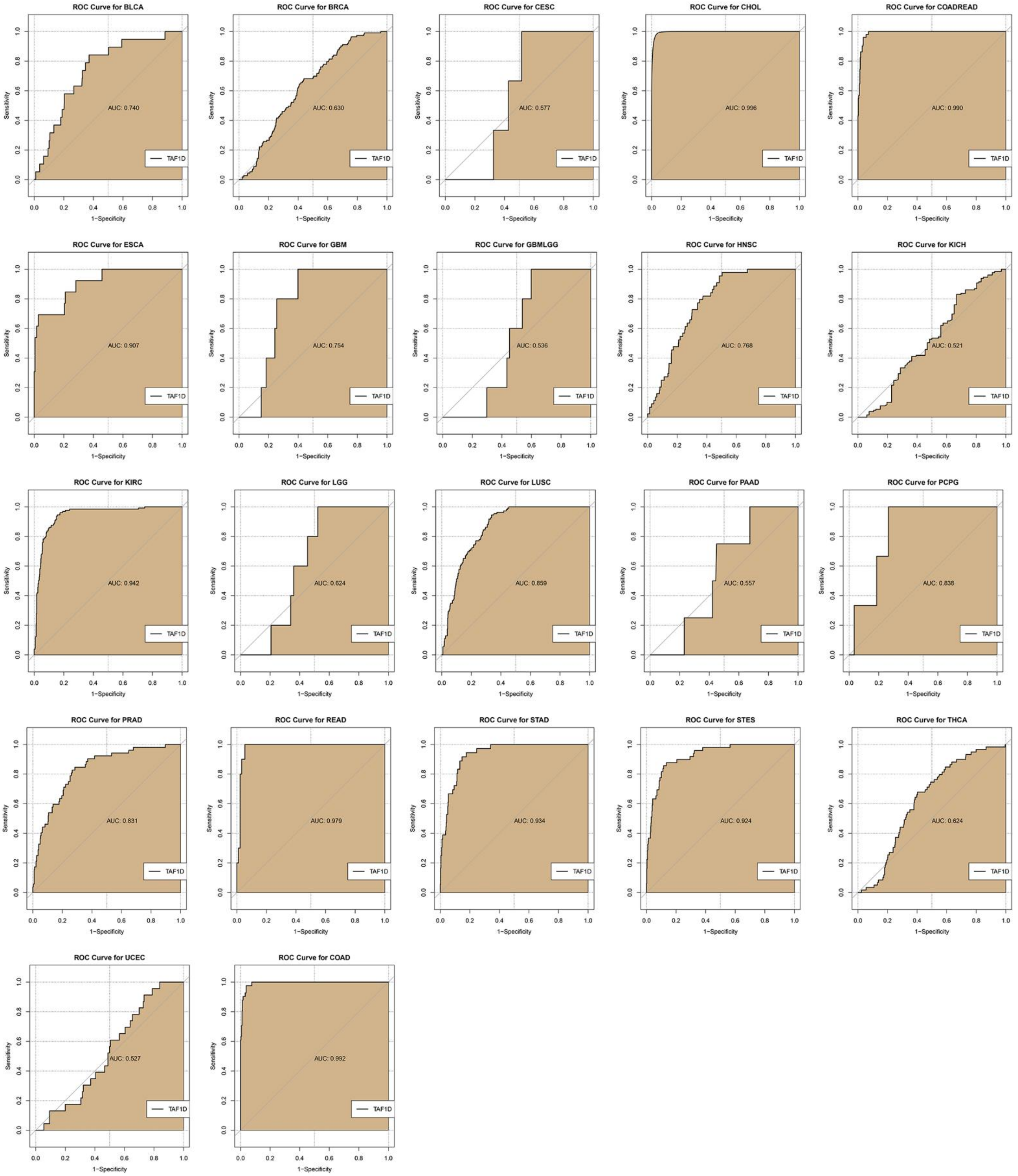

Additional Fig. Supplementary 2. ROC curves of TAF1D in 22 different malignant cancer based on the TCGA pan-caner dataset.

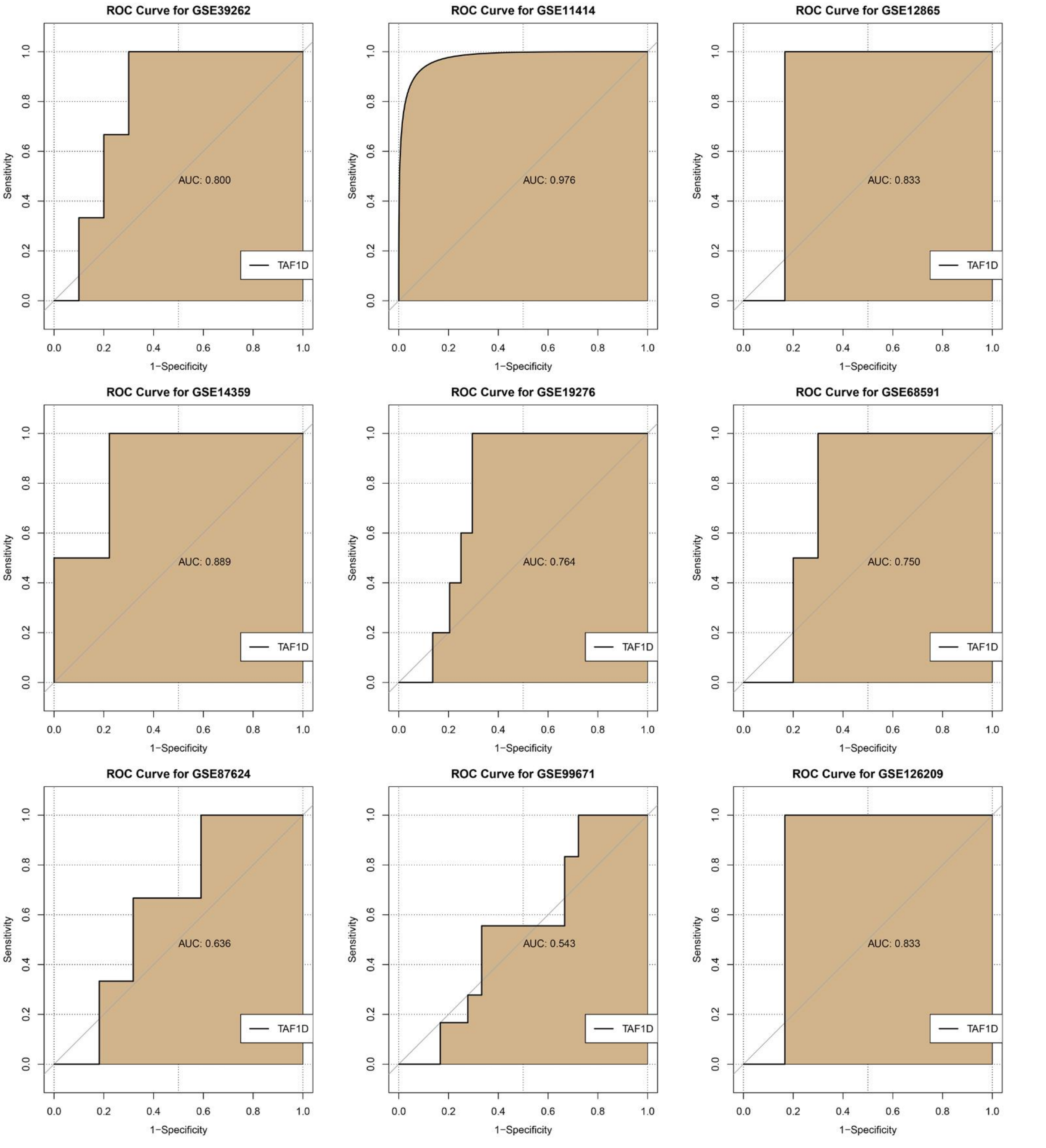

Additional Fig. Supplementary 3. ROC curves of TAF1D in OS based on GEO datasets.

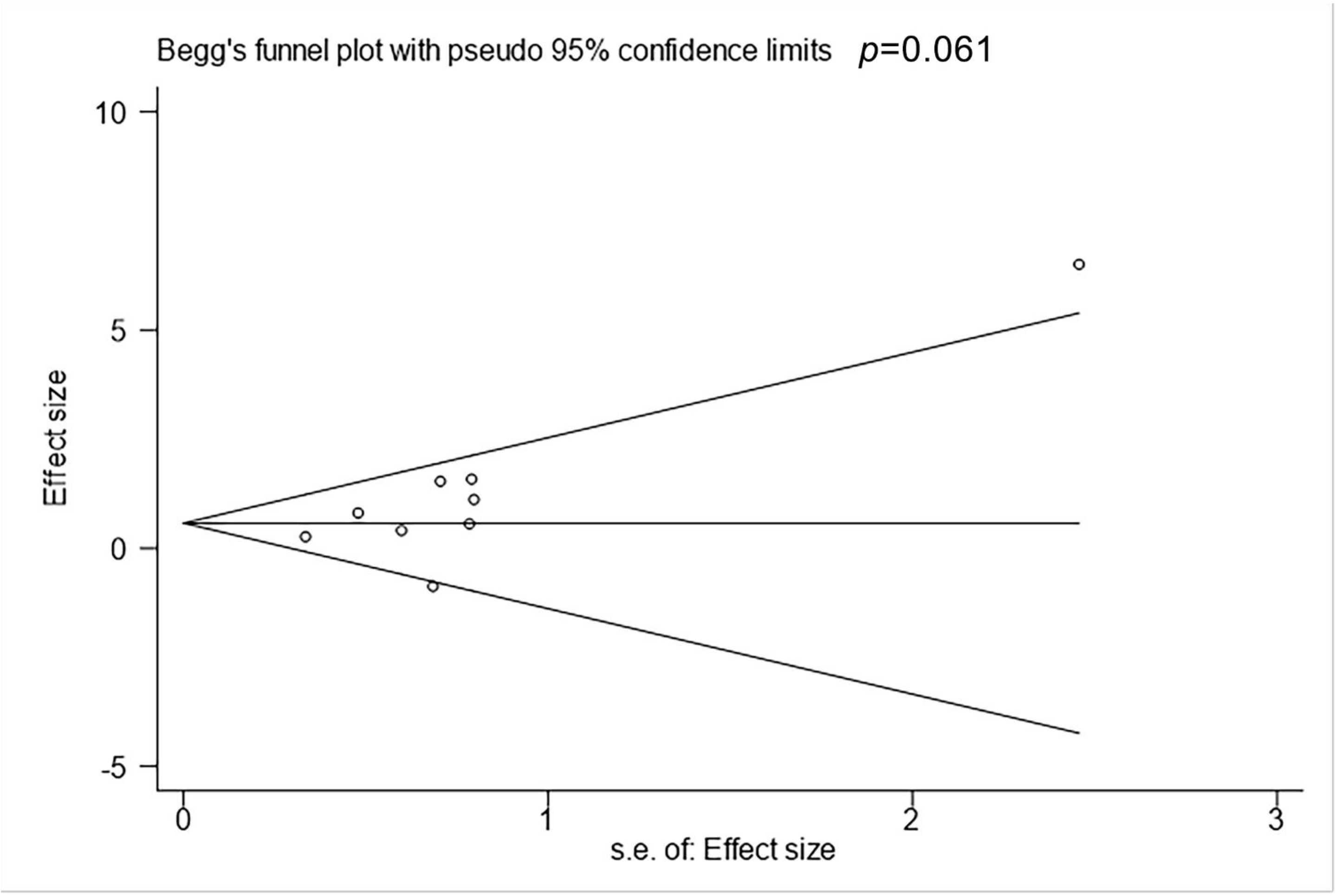

Additional Fig. Supplementary 4. Begg’s test showing no publication bias.

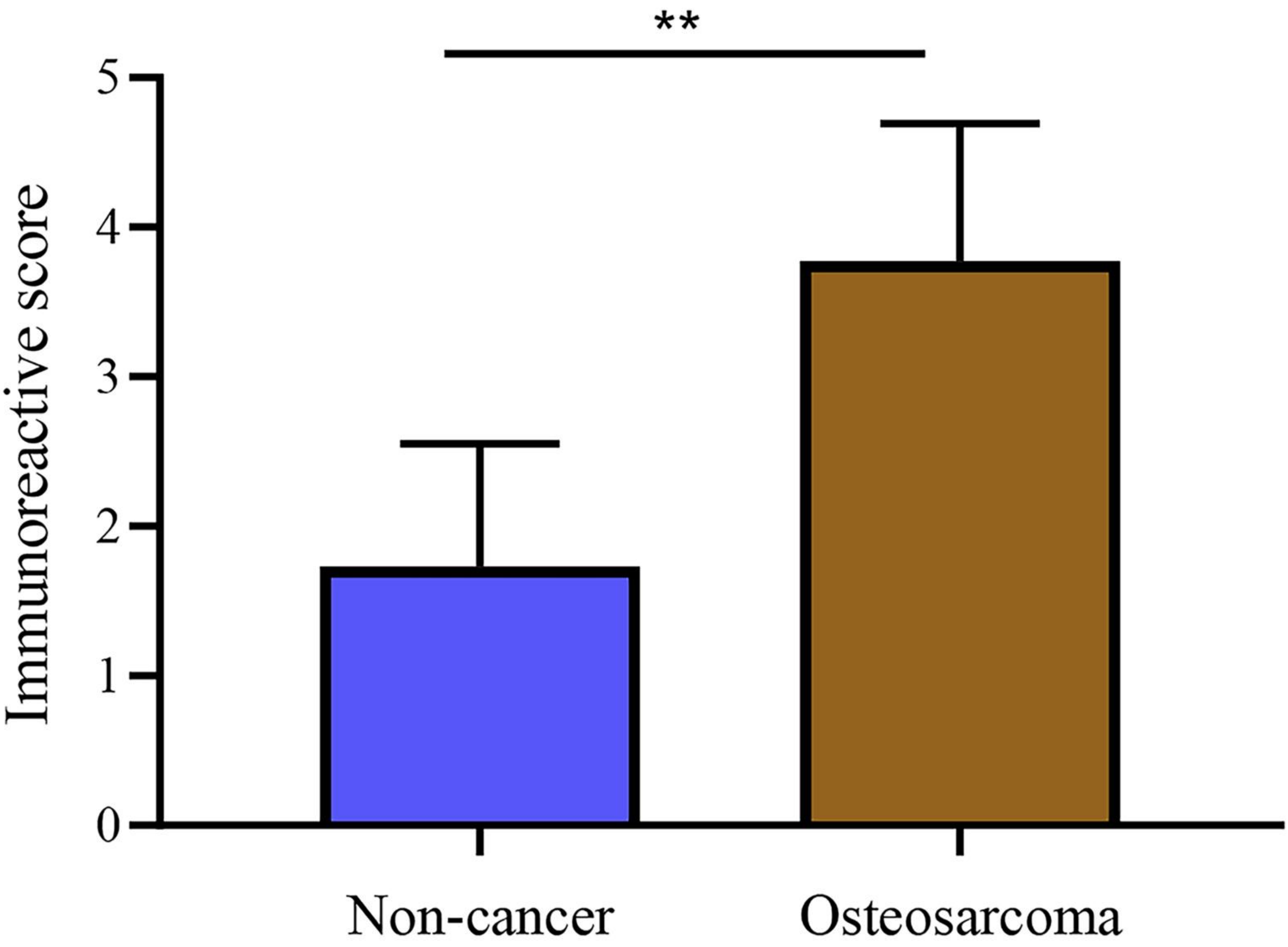

Additional Fig. Supplementary 5. Bar chart of TAF1D protein levels in OS. *p <0.05; **p <0.01; ***p<0.001; ****p<0.0001.

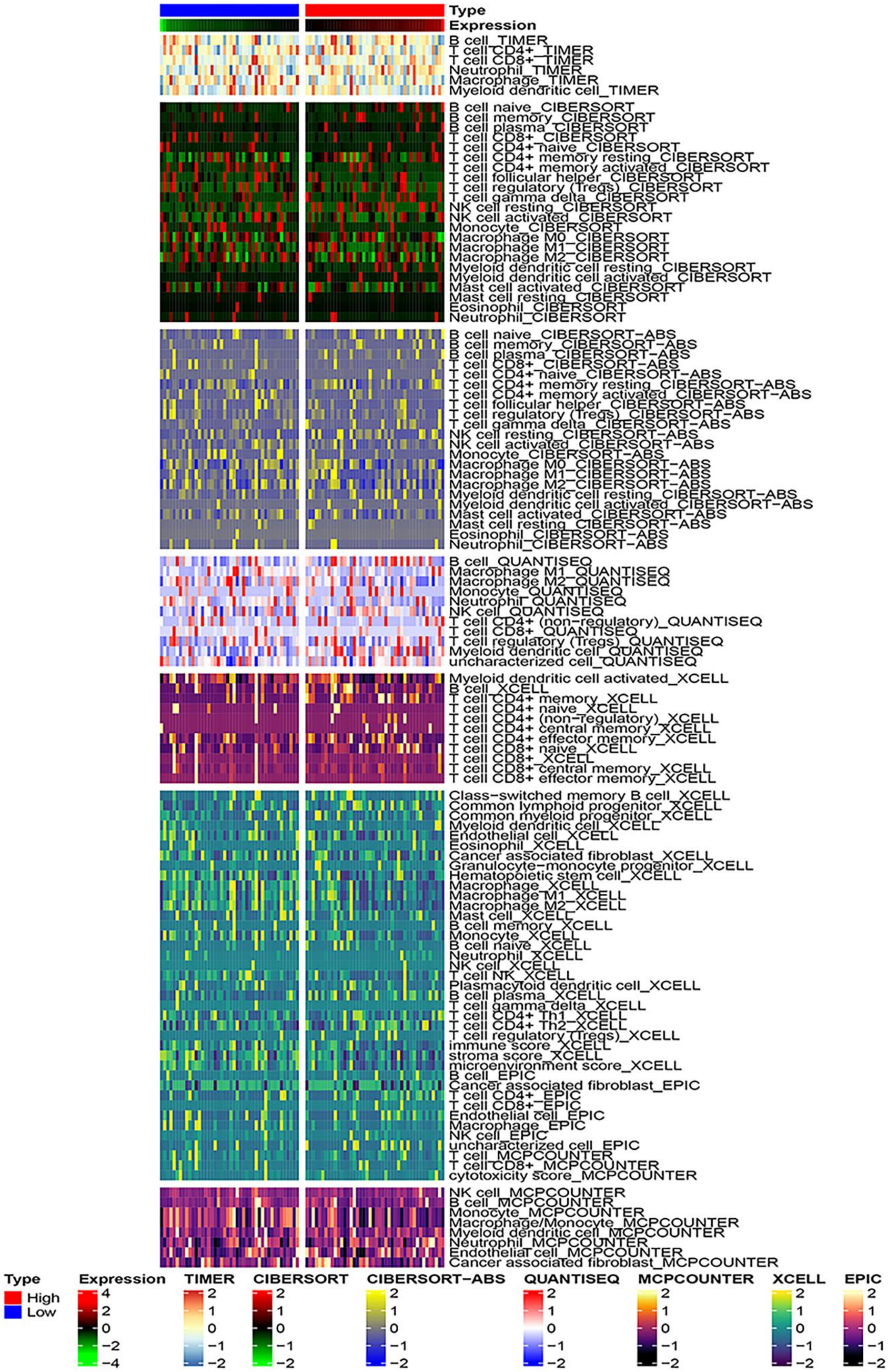

Additional Fig. Supplementary 6. According to the median of TAF1D expression level, the relationship between TAF1D and various immune cell infiltration levels was displayed in the form of heatmap.

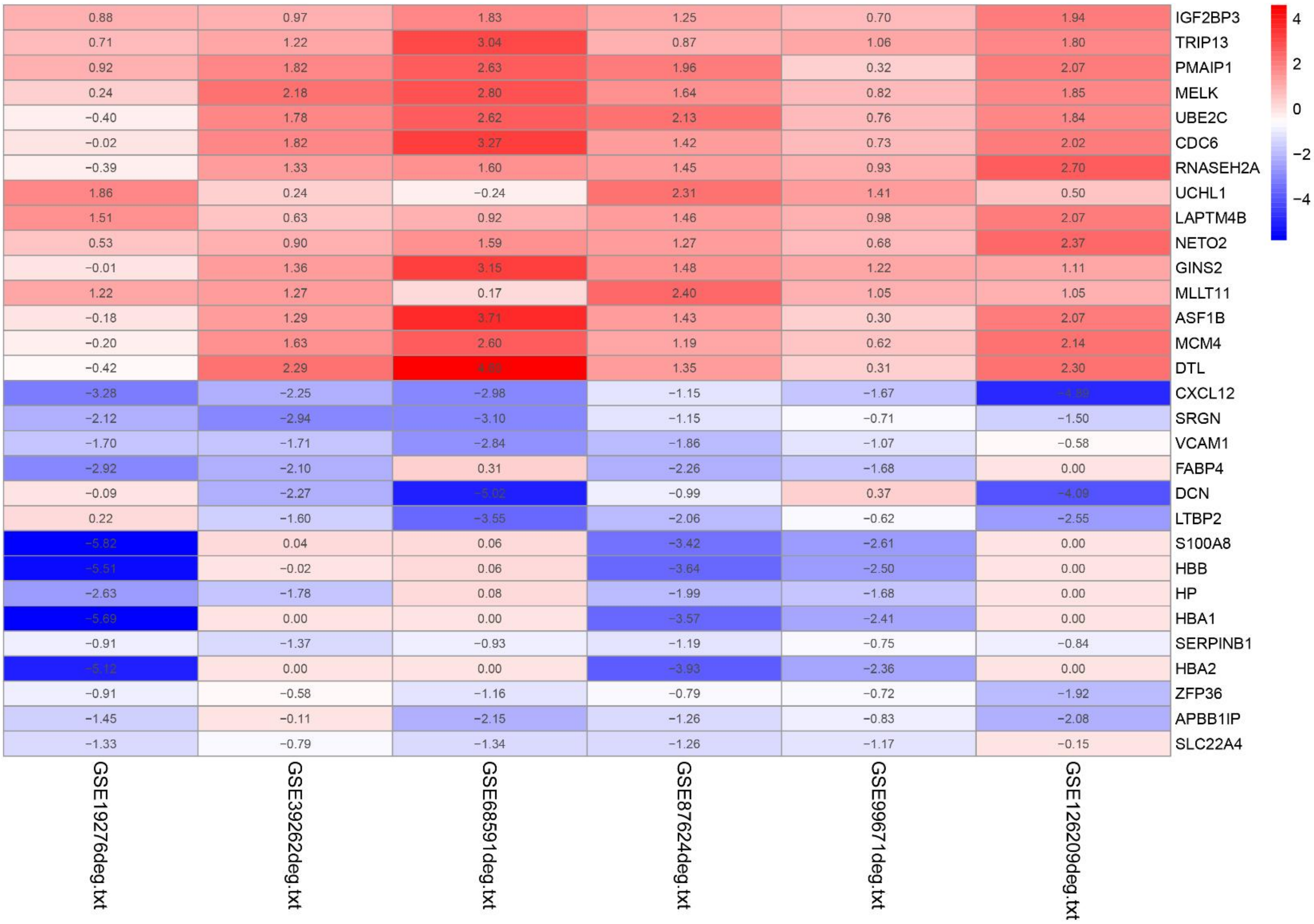

Additional Fig. Supplementary 7. Identification of DEGs of GEO datasets using RRA. The heatmap of the top 15 up- and down-regulated DEGs in the integrated GEO datasets analysis. Red represents up-regulated genes, blue represents down-regulated genes.

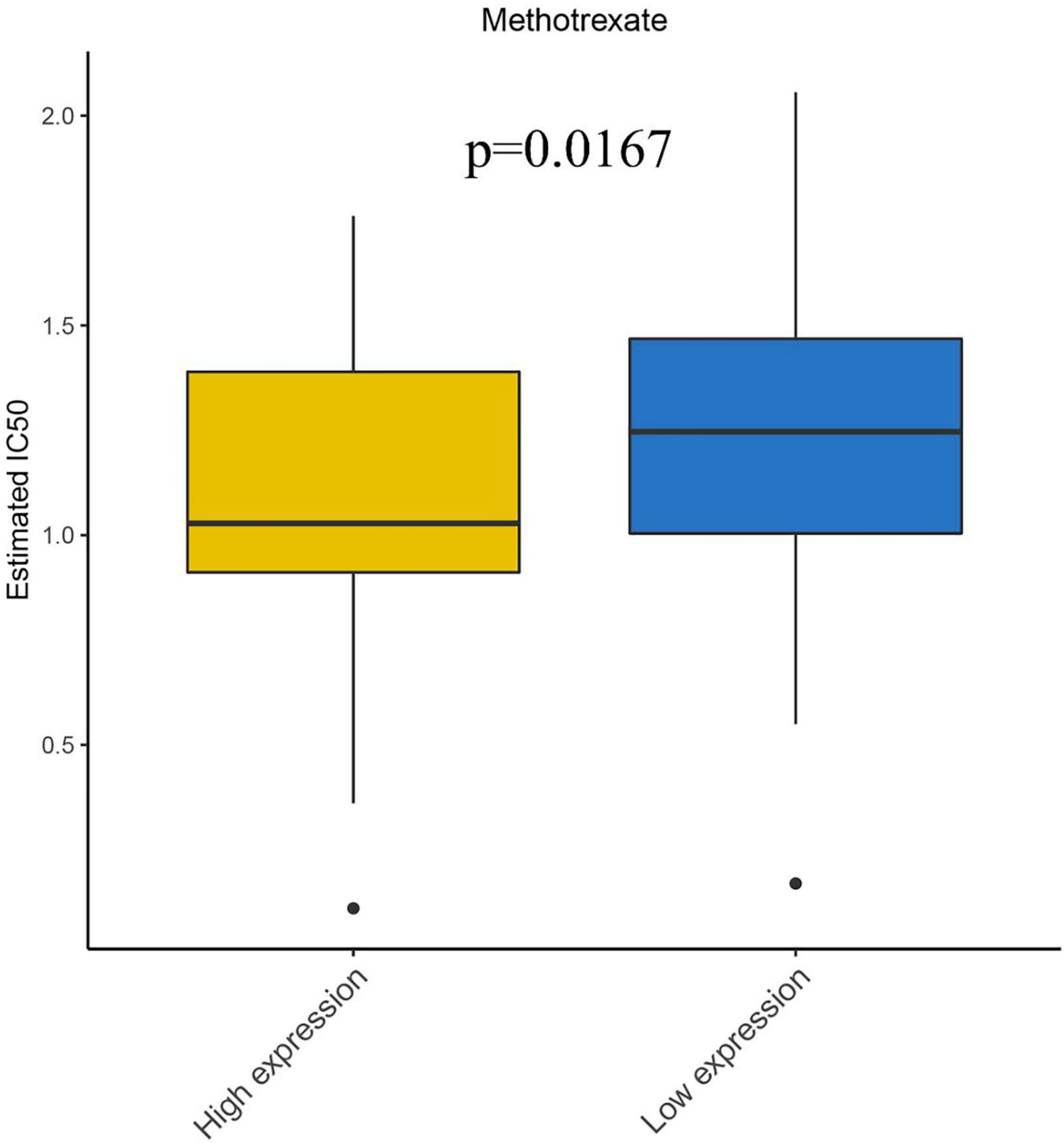

Additional Fig. Supplementary 8. Methotrexate showed significant difference between high- and low-TAF1D groups.

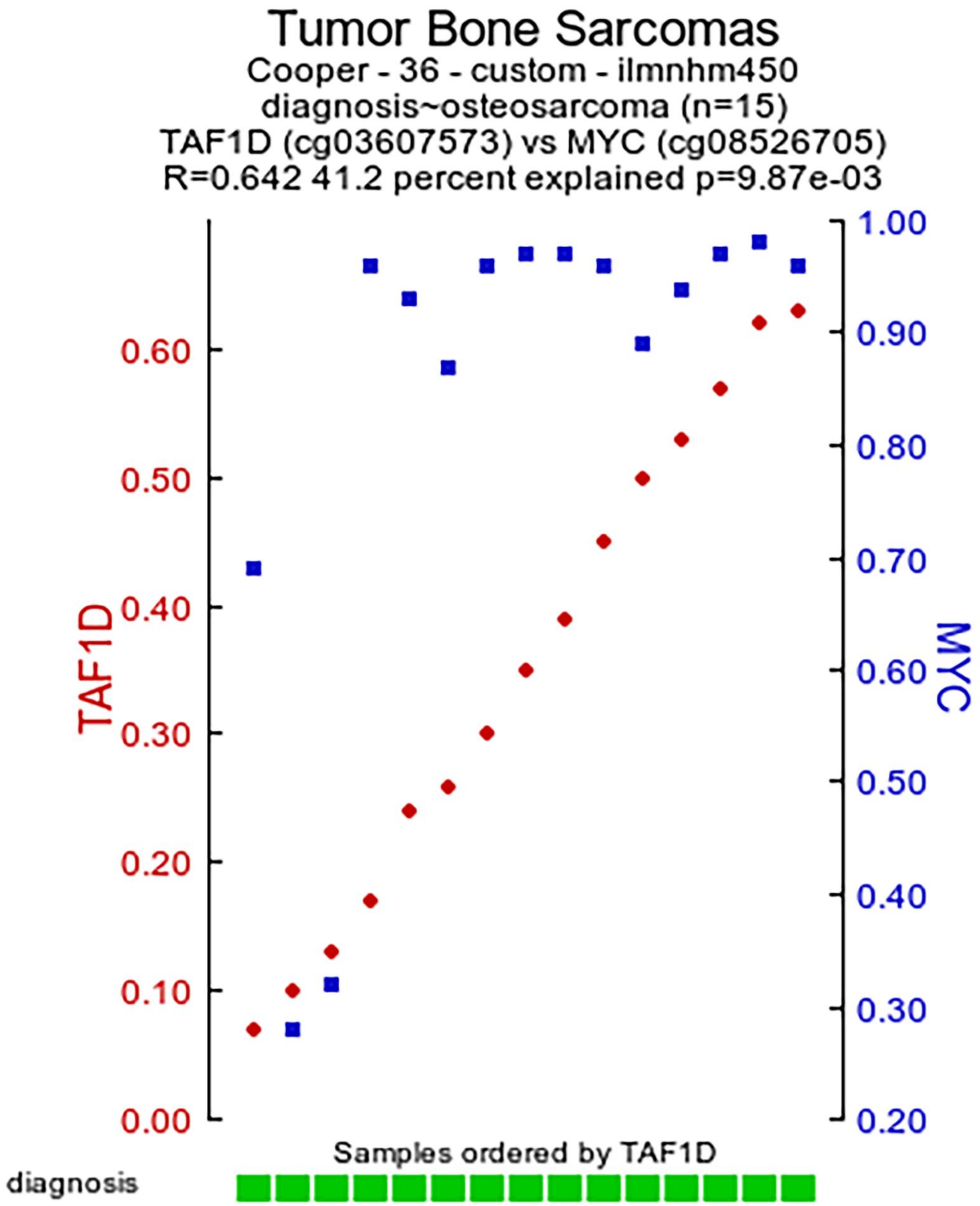

Additional Fig. Supplementary 9. Correlation between methylation level of TAF1D and MYC.

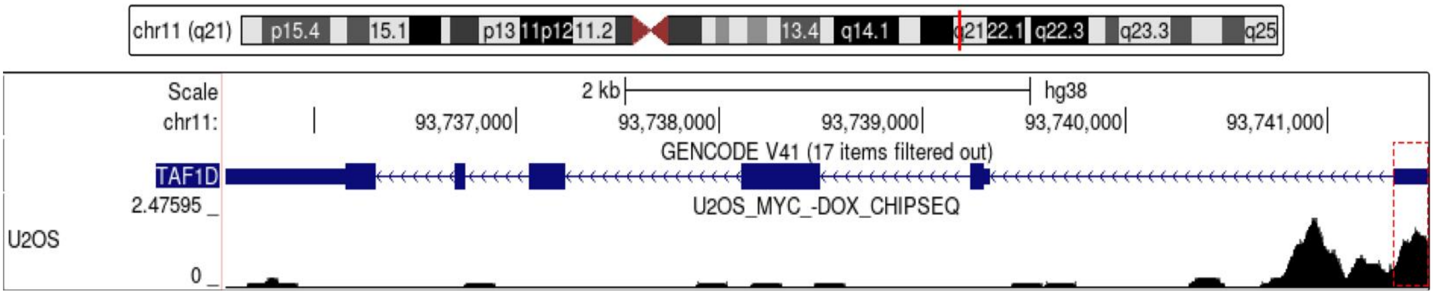

Additional Fig. S10. TAF1D had binding peaks of MYC in the initial region of translation.

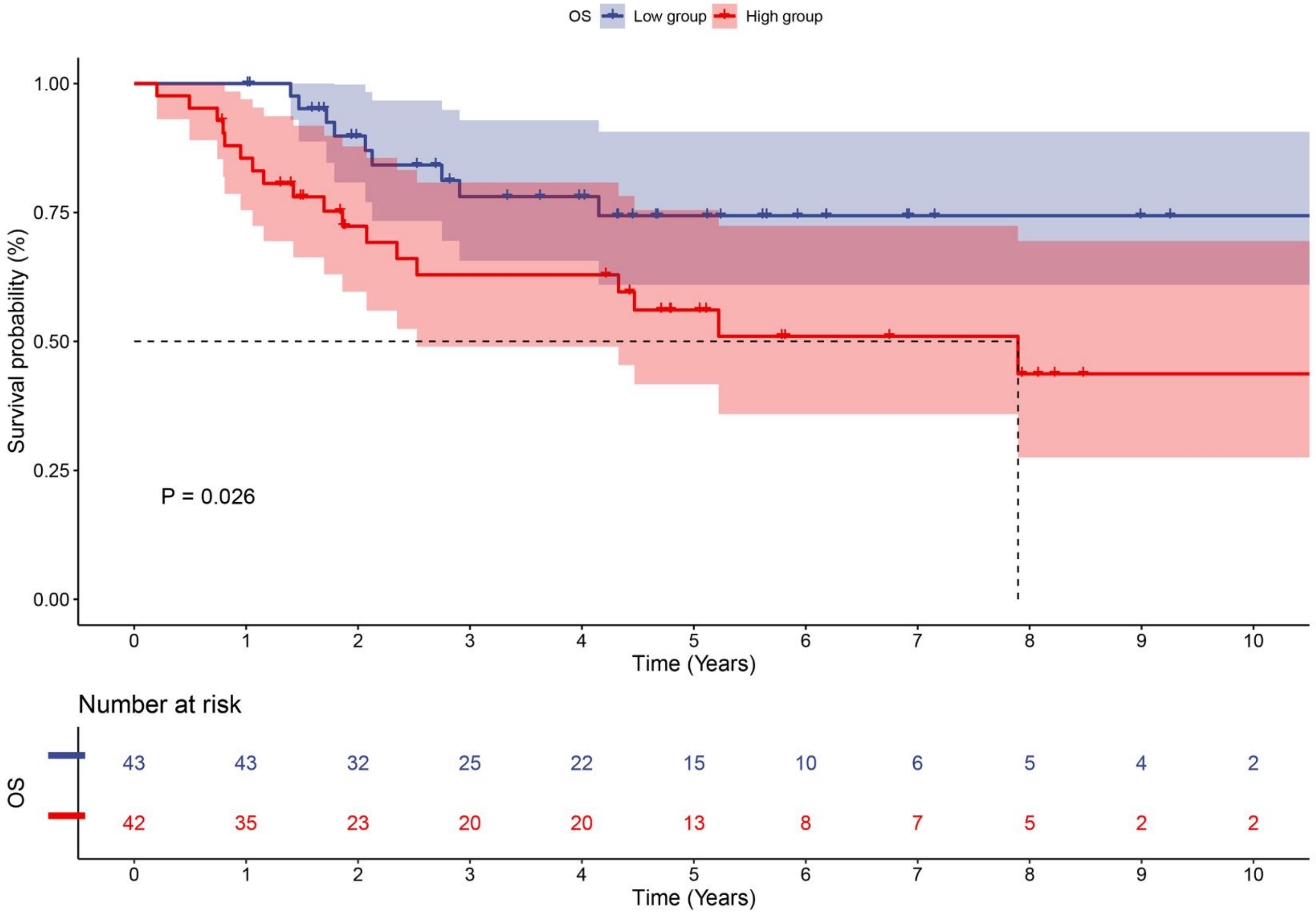

Additional Fig. Supplementary 11. MYC high expression was related to shorter survival time of OS patients.

Supplementary Table 1. The 35 significantly upregulated DEGs through the RRA algorithm.

Name	*p*-value	adj p-value	log2FC
IGF2BP3	8.70E-11	1.98E-06	1.26E+00
TRIP13	3.25E-09	7.42E-05	1.45E+00
PMAIP1	8.31E-09	1.89E-04	1.62E+00
MELK	8.94E-09	2.04E-04	1.59E+00
UBE2C	1.05E-08	2.39E-04	1.46E+00
CDC6	1.50E-08	3.42E-04	1.54E+00
RNASEH2A	2.36E-08	5.39E-04	1.27E+00
UCHL1	2.78E-08	6.33E-04	1.01E+00
LAPTM4B	3.14E-08	7.16E-04	1.26E+00
NETO2	7.61E-08	1.74E-03	1.23E+00
GINS2	1.23E-07	2.81E-03	1.38E+00
MLLT11	2.62E-07	5.98E-03	1.19E+00
ASF1B	2.95E-07	6.72E-03	1.44E+00
MCM4	2.96E-07	6.75E-03	1.33E+00
DTL	3.53E-07	8.05E-03	1.74E+00
ISG15	3.92E-07	8.94E-03	1.07E+00
MCM3	4.56E-07	1.04E-02	1.56E+00
KPNA2	4.75E-07	1.08E-02	1.08E+00
ATAD2	5.58E-07	1.27E-02	1.54E+00
CDC45	5.58E-07	1.27E-02	1.30E+00
MCM2	7.69E-07	1.75E-02	1.36E+00
PBK	8.26E-07	1.88E-02	1.99E+00
ZWINT	8.41E-07	1.92E-02	1.26E+00
APOBEC3B	9.66E-07	2.20E-02	1.28E+00
MAD2L1	1.16E-06	2.63E-02	1.34E+00
MAGEA1	1.17E-06	2.68E-02	1.18E+00
PRAME	1.19E-06	2.70E-02	1.84E+00
AURKB	1.20E-06	2.73E-02	1.31E+00
CDKN3	1.26E-06	2.87E-02	1.57E+00
TOP2A	1.42E-06	3.23E-02	1.79E+00
RAD51AP1	1.48E-06	3.37E-02	1.44E+00
CDC20	1.59E-06	3.62E-02	1.58E+00
CHAF1B	1.61E-06	3.68E-02	1.17E+00
TMEM97	1.62E-06	3.70E-02	1.05E+00
PRC1	2.17E-06	4.96E-02	1.32E+00

DEGs: differentially expressed genes; RRA: RobustRankAggreg.
